# Metabolic score for insulin resistance (METS-IR) predicts all-cause and cardiovascular mortality in the general population: evidence from NHANES 2001–2018

**DOI:** 10.1186/s12933-024-02334-8

**Published:** 2024-07-10

**Authors:** Mingxuan Duan, Xi Zhao, Shaolin Li, Guangrui Miao, Linpeng Bai, Qingyang Zhang, Wenxuan Yang, Xiaoyan Zhao

**Affiliations:** Henan Key Laboratory of Hereditary Cardiovascular Diseases, Department of Cardiology, Cardiovascular Center, The First Affiliated Hospital of Zhengzhou University, Zhengzhou, Henan China

**Keywords:** Metabolic score for insulin resistance, Triglyceride-glucose index, Mortality, Boruta algorithm, Insulin resistance, NHANES

## Abstract

**Background:**

The prevalence of obesity-associated insulin resistance (IR) is increasing along with the increase in obesity rates. In this study, we compared the predictive utility of four alternative indexes of IR [triglyceride glucose index (TyG index), metabolic score for insulin resistance (METS-IR), the triglyceride/high-density lipoprotein cholesterol (TG/HDL-C) ratio and homeostatic model assessment of insulin resistance (HOMA-IR)] for all-cause mortality and cardiovascular mortality in the general population based on key variables screened by the Boruta algorithm. The aim was to find the best replacement index of IR.

**Methods:**

In this study, 14,653 participants were screened from the National Health and Nutrition Examination Survey (2001–2018). And TyG index, METS-IR, TG/HDL-C and HOMA-IR were calculated separately for each participant according to the given formula. The predictive values of IR replacement indexes for all-cause mortality and cardiovascular mortality in the general population were assessed.

**Results:**

Over a median follow-up period of 116 months, a total of 2085 (10.23%) all-cause deaths and 549 (2.61%) cardiovascular disease (CVD) related deaths were recorded. Multivariate Cox regression and restricted cubic splines analysis showed that among the four indexes, only METS-IR was significantly associated with both all-cause and CVD mortality, and both showed non-linear associations with an approximate “U-shape”. Specifically, baseline METS-IR lower than the inflection point (41.33) was negatively associated with mortality [hazard ratio (HR) 0.972, 95% CI 0.950–0.997 for all-cause mortality]. In contrast, baseline METS-IR higher than the inflection point (41.33) was positively associated with mortality (HR 1.019, 95% CI 1.011–1.026 for all-cause mortality and HR 1.028, 95% CI 1.014–1.043 for CVD mortality). We further stratified the METS-IR and showed that significant associations between METS-IR levels and all-cause and cardiovascular mortality were predominantly present in the nonelderly population aged < 65 years.

**Conclusions:**

In conjunction with the results of the Boruta algorithm, METS-IR demonstrated a more significant association with all-cause and cardiovascular mortality in the U.S. population compared to the other three alternative IR indexes (TyG index, TG/HDL-C and HOMA-IR), particularly evident in individuals under 65 years old.

## Background

In recent years, obesity has increasingly become a widespread epidemic as quality of life continues to improve. Obesity rates [body mass index (BMI) > 30] and severe obesity rates (BMI > 35) are projected to rise to 50% and 25%, respectively, across the United States by 2030. Along with this increase in obesity rates, the prevalence of obesity-related insulin resistance (IR) and cardiovascular disease (CVD) will also increase [1, 2]. Clinically, IR, known as syndrome X, or insulin resistance syndrome, is a state of reduced sensitivity and responsiveness to the action of insulin that usually occurs several years before the onset of diabetes and has been shown to increase the risk of CVD [3–5]. Studies have shown that the state of hyperinsulinemia caused by IR can accelerate the production of fatty acids, impede the normal action of insulin and may trigger early atherosclerosis, atherosclerotic dyslipidemia, dysglycemia and blood pressure abnormalities [6]. Long-term metabolic disorder will increase the cardiovascular disease mortality and even all-cause mortality of patients. Therefore, early recognition of insulin resistance status is crucial for early treatment.

The hyperinsulinemic-euglycemic clamp technique is considered the gold standard for the assessment of IR, but due to its high assay cost and complex procedure, the method is limited to small studies and not used in large epidemiologic investigations [7]. The homeostasis model assessment of insulin resistance (HOMA-IR) is widely used in the assessment of IR due to its non-invasiveness and simplicity. However, the assessment efficacy of this model varies by population race and shows some limitations in patients treated with insulin or in patients with β-cell insufficiency [8, 9]. In addition, calculation of HOMA-IR relies on laboratory measurement of fasting insulin levels, which is often difficult to achieve in resource-limited countries, limiting its popularity in daily clinical practice. Therefore, there is a need for a less costly and more readily available indicator to assess IR more broadly and easily. Currently, the alternative indexes of IR commonly used in clinical practice are triglyceride glucose index (TyG index), metabolic score for Insulin resistance (METS-IR), the triglyceride /high-density lipoprotein cholesterol (TG/HDL-C) ratio and homeostatic model assessment of insulin resistance (HOMA-IR). The TyG index is inexpensive to calculate and easy to obtain, and is recognized as a time-saving and relatively simple marker of IR [10]. Multiple studies have shown that it performs consistently or better than HOMA-IR in assessing IR [11, 12]. Therefore, a large number of studies have been conducted to explore the relationship between TyG index and cardiovascular diseases and their prognosis. Some studies have reported that TyG index is significantly associated with all-cause mortality and cardiovascular mortality in the general population, especially in people under 65 years of age [13]. However, some studies have reported inconsistent results, stating that there is no significant relationship between the TyG index and all-cause or cardiovascular mortality [14]. The controversy surrounding the TyG index has somewhat limited its clinical application. In addition, there are fewer studies on other indexes of IR besides the TyG index. The existence of alternative indexes of IR that are better predictors of all-cause or cardiovascular mortality is an issue that needs to be focused on and resolved, which is essential to find the best alternative index of IR and to promote its clinical application.

Boruta algorithm is a supervised classification feature selection method based on random forests, which minimizes the error of the random forest model and ultimately forms a subset of the minimum optimal features [15, 16]. Most studies rely on clinical significance and experience in selecting variables for inclusion in multivariate Cox proportional risk models. In this study, we use the results of Boruta algorithm in conjunction with practical clinical significance to screen the variables to be included in multivariate Cox regression.

In this study, we included four replacement indexes of IR, TyG index, METS-IR, TG/HDL-C, and HOMA-IR, with the aim of comparing the predictive effects of these four indices on all-cause mortality and cardiovascular mortality in the general population, searching for the optimal replacement indices for insulin resistance, and identifying high-risk groups for insulin resistance.

## Methods

### Study population and design

**Fig. 1:**
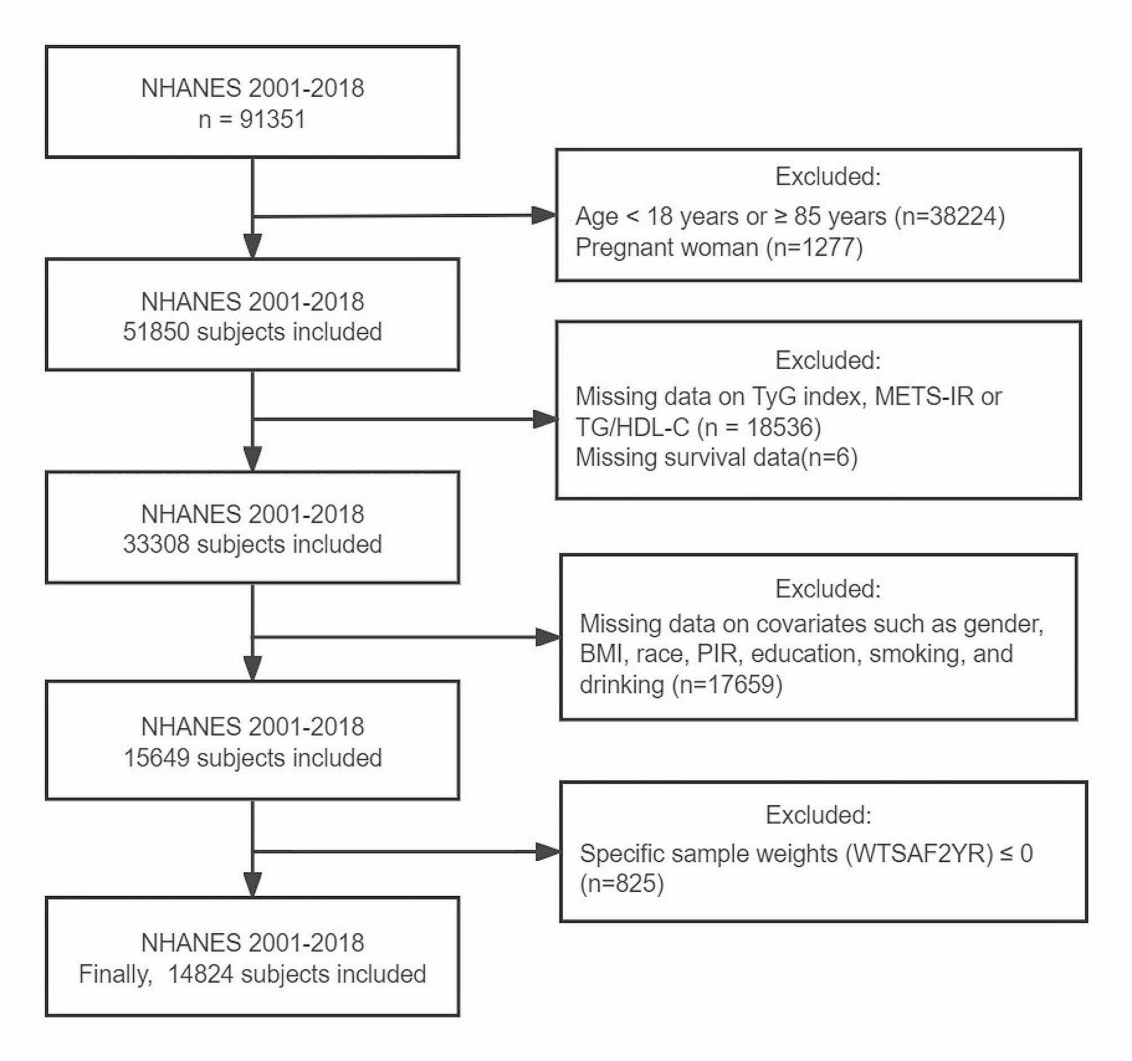
Flow diagram of patient selection. *TyG index* triglyceride glucose index, *METS-IR* metabolic score for Insulin resistance, *TG* triglyceride, *HDL-C* high-density lipoprotein-cholesterol, *HOMA-IR* homeostatic model assessment of insulin resistance, *BMI* body mass index, *PIR* the ratio of family income to poverty

The National Health and Nutrition Examination Survey (NHANES) is a major scientific program conducted by the National Center for Health Statistics (NCHS) of the Centers for Disease Control and Prevention (CDC). The survey is dedicated to assessing the health and nutritional status of the non-institutionalized population living in the United States, including adults and children, in order to provide a comprehensive picture of the health of Americans. Data are collected by NCHS every two years. NHANES received approval from the NCHS Research Ethics Review Board. Datasets generated and analyzed by NHANES are available on the NHANES website (http://www.cdc.gov/nchs/nhanes.htm). We downloaded NHANES data for nine cycles from 2001 to 2018 with the aim of investigating the association between the four alternative indexes of IR (TyG index, METS-IR, TG/HDL-C, and HOMA-IR) and all-cause and cardiovascular mortality. A total of 91,351 participants were surveyed. The following patients were excluded from the study: (1) Patients under 18 years of age or 85 years of age and older. (2) Pregnant patients. (3) Patients who lacked a calculated index related to the IR replacement indexes such as fasting triglycerides, high-density lipoprotein, fasting plasma glucose (FPG) and fasting serum insulin (FINS). (4) Patients lacking prognostic data. (5) Patients without important covariates such as gender, body mass index (BMI), race, poverty income ratio (PIR), education, smoking and drinking. (6) Patients with fasting subsample weights (WTSAF2YR) ≤ 0. The final study population consisted of 14,653 participants. The patient selection process is shown in Fig. 1.

### Insulin resistance replacement index

Four IR replacement indexes were included in this study, namely TyG Index, METS-IR, TG/HDL-C and HOMA-IR. TyG index = Ln[triglycerides (mg/dL) × glycemia (mg/dL)/2] [17]. METS-IR = Ln[2 × glycemia (mg/dL) + triglycerides (mg/dL)] × BMI/Ln HDL-C (mg/dL) [18]. TG/HDL-C calculated as triglycerides (mg/dL)/HDL-C (mg/dL) [19]. HOMA-IR was calculated using the formula FPG (mmol/L) × FINS (mIU/L) /22.5 [20].

### Determination of mortality rates

The primary endpoint of this study was all-cause mortality and the secondary endpoint was cardiovascular mortality. To determine the mortality status of the follow-up population, we employed the NHANES public-use linked mortality file as of December 31, 2019, which was correlated to the NCHS and the National Death Index (NDI) through a probabilistic matching algorithm. Follow-up began on the date of the NHANES interview and ended on the date of death or the end of follow-up (December 31, 2019). In addition, cardiovascular death was defined as deaths due to cardiovascular disease according to the International Statistical Classification of Diseases, 10th Revision (ICD-10) and the NCHS classifying heart diseases (054-068).

### Assessment of covariates

NHANES personnel used questionnaires in the household interviews, through which a variety of demographic and health-related information was collected, including age, gender, race, household income, education, smoking and drinking status. Race was categorized as Mexican American, other Hispanic, Non-Hispanic White, Non-Hispanic Black or Other. Household income was classified as low income, middle income and high income, based on the family PIR (< 1.0, 1.0–3.0, > 3.0), respectively. Education level was classified as less than high school, high school or equivalent, or college or above. Drinking status was recorded as never drinker (no more than 12 drinks in a lifetime), former drinker (no more than 12 drinks in the past year) and current drinker (greater than or equal to 12 drinks in the past year). Smoking status was recorded as never smoker (less than 100 cigarettes in a lifetime), former smoker (more than 100 cigarettes in a lifetime and had quit smoking for more than 1 year at the time of the survey) and current smoker (more than 100 cigarettes in lifetime and still smoking or quit less than 1 year ago at the time of the survey).

NHANES personnel measured participants’ height, weight, BMI, and blood pressure at the Mobile Examination Center (MEC). BMI was calculated as weight in kilograms divided by height in meters squared. BMI was divided into four groups based on the World Health Organization’s standardized thresholds, as follows Underweight: BMI < 18.5 kg/m^2^; Normal: 18.5 ≤ BMI < 25.0 kg/m^2^; Overweight: 25.0 ≤ BMI < 30.0 kg/m^2^; Obesity: BMI ≥ 30.0 kg/m^2^ [21]. In NHANES, serum specimens were collected as part of the laboratory tests to measure clinical markers such as glycosylated hemoglobin (HbA1C), FPG, low-density lipoprotein cholesterol (LDL-C), HDL-C, total cholesterol (TC), TG and serum creatinine (Scr) at baseline.

The comorbidities analyzed in this study comprised hypertension, diabetes, hyperlipidemia, CVD and metabolic syndrome (MetS). Participants’ blood pressures were obtained at the MEC, and details of the blood pressure measurements can be found on the NHANES website (http://www.cdc.gov/nchs/nhanes.htm, accessed on 11 November 2022). Hypertension was defined as the fulfillment of any of the following conditions: mean systolic blood pressure (SBP) ≥ 140 mmHg or mean diastolic blood pressure (DBP) ≥ 90 mmHg on three consecutive measurements in a calm state, self-reported hypertension, or the use of prescription antihypertensive medications. This criterion is based on the guidelines of the International Society of Hypertension, in which 140/90 mmHg is the threshold for determining hypertension [22]. The definition of diabetes includes the following criteria, one of which was sufficient for diagnosis: FPG ≥ 7.0 mmol/L (126 mg/dL), 2-h postprandial plasma glucose ≥ 11.1 mmol/L (200 mg/dL), HbA1C ≥ 6.5%, self-reported diabetes mellitus and insulin or oral hypoglycemic medication use [23]. Hyperlipidemia was defined in conjunction with the National Cholesterol Education Program (NCEP) Adult Treatment Third Edition (ATP 3) criteria: total cholesterol ≥ 200 mg/dL, triglycerides ≥ 150 mg/dL, HDL-C ≤ 40 mg/dL in men and ≤ 50 mg/dL in women, and LDL-C ≥ 130 mg/dL, self-reported cholesterol levels high or using cholesterol-lowering drugs [24]. The diagnosis of CVD was confirmed by asking participants “Has a doctor or other health expert ever informed you that you have congestive heart failure (CHF)/coronary heart disease (CHD)/angina pectoris/myocardial infarction (MI)/stroke?” If a person answered “yes” to any of these questions, they were considered to have CVD [4]. Metabolic syndrome was defined as follows: (1) waist circumference (WC) ≥ 94 cm in men or ≥ 80 cm in women; (2) SBP/DBP ≥ 130/85 mmHg or use of antihypertensive medications; (3) TG ≥ 150 md/dL or use of lipid-lowering medications; (4) HDL-C < 40 mg/dL in men or < 50 mg/dL in women; (5) FPG ≥ 100 mg/dL or diagnosed diabetes. Metabolic syndrome was diagnosed by meeting any two or more of conditions (2–5) on the basis of condition (1) [25, 26].

### Statistical analysis

Due to the complexity of the NHANES sampling design, analyzing NHANES data requires that sample weights, clustering and stratification be incorporated into all analyses. Sampling weights were considered for all analyses in this study. According to the NHANES analysis guidelines, new weights were calculated by dividing the weights for the 2-year cycle by 9. We used the four alternative indexes of insulin resistance, TyG index, METS-IR, TG/HDL-C and HOMA-IR, as independent variables and stratified the study population characteristics and laboratory indexes according to their quartiles, and compared the baseline characteristics of the groups using one-way ANOVA for continuous variables and Pearson chi-square test for categorical variables. Continuous variables were expressed as mean and standard deviation (SD), and categorical variables were expressed as frequency and percentage. In addition, we divided the study cohort into survival and death groups based on patient prognosis and compared the baseline characteristics of the populations in each group with the same statistical methods as above.

Feature selection is an important step in model construction, and in this study, we used the Boruta algorithm, a supervised categorical feature selection method, to pinpoint all relevant features. Next, Cox proportional risk regression models were used to estimate hazard ratios (HRs) and their corresponding 95% confidence intervals (CIs) to test the relationship between the IR replacement indexes and all-cause and cardiovascular mortality. Model 1 represents a univariate Cox regression analysis. Model 2 was adjusted for age, sex and race. Model 3 was adjusted for all-cause mortality and cardiovascular mortality separately based on the specific results of the Boruta algorithm. Nonlinear correlations between the IR replacement index and mortality were explored using restricted cubic spline (RCS) regression models. If the relationship was linear, we selected the point at which the HR was 0 as the “inflection point”. We used two segmented Cox proportional risk models on either side of the inflection point to examine the relationship between the insulin resistance replacement indexes and mortality.

We further stratified the analysis of significant covariates to consider potential influences. In this study, we stratified analyses according to age (< 65 or ≥ 65 years), sex, BMI, education, smoking status, hypertension, diabetes mellitus and metabolic syndrome. R (V 4.3.1) was used for all statistical analyses. A P value of < 0.05 was determined to be statistically significant.

## Results

### Baseline characteristics of study participants

**Fig. 2:**
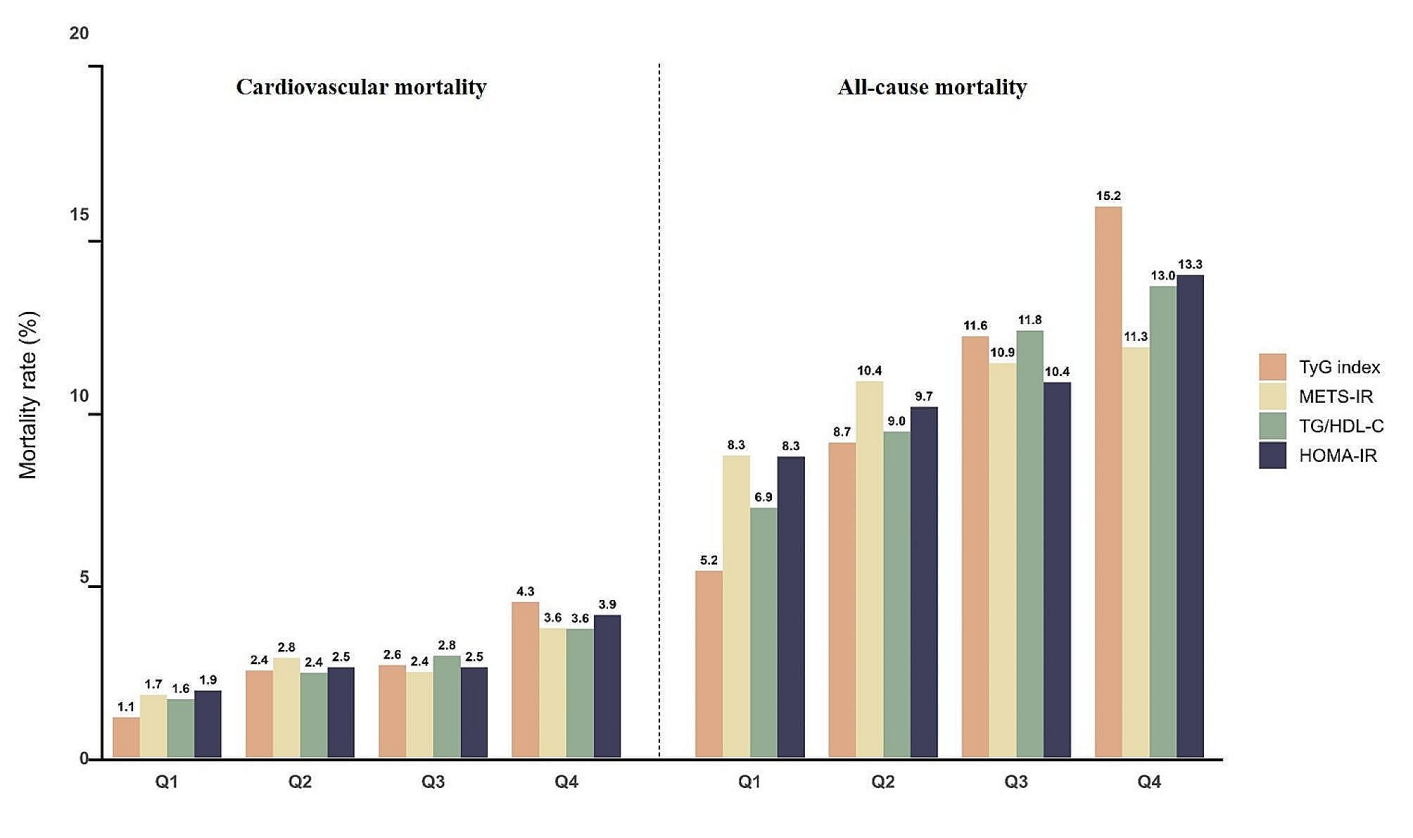
All-cause mortality and cardiovascular mortality bar graphs based on quartiles of TyG index, METS-IR, TG/HDL-C and HOMA-IR stratification. *TyG index* triglyceride glucose index, *METS-IR* metabolic score for Insulin resistance, *TG* triglyceride, *HDL-C* high-density lipoprotein-cholesterol, *HOMA-IR* homeostatic model assessment of insulin resistance

Table 1 shows the baseline characteristics of the cohort study participants (14,653) stratified by TyG index, METS-IR, TG/HDL-C and HOMA-IR quartiles. The mean age of the participants was 46 years, of which 49.82% were male. The mean TyG index, METS-IR, TG/HDL-C and HOMA-IR of the enrolled patients were 8.63 ± 0.66, 42.92 ± 12.54, 2.97 ± 3.83 and 3.39 ± 5.23, respectively. During 1,743,607 person-months of follow-up (median follow-up of 116 months), there were 2085 (10.23%) incident cases of all-cause mortality and 549 (2.61%) incident cases of cardiovascular mortality. Laboratory characteristics at baseline based on TyG index, METS-IR, TG/HDL-C and HOMA-IR quartiles are shown in Table 2. The baseline and laboratory characteristics of the enrolled patients showed approximately the same trend across the four indexes, with patients in the higher-scoring group tending to be older, male, obese, with a medium or lower household income, with a lower level of education, former alcohol drinkers and smokers compared to those in the lower-scoring group, and exhibiting a higher prevalence of comorbidities, including hypertension, diabetes mellitus, hyperlipidemia, CVD and metabolic syndrome (*P* < 0.001). In addition, the group with higher scores was associated with higher SBP, DBP and blood indices, including HbA1C, fasting serum insulin (FINS), FPG, TG, Uric acid, blood urea nitrogen (BUN), alanine transaminase (ALT), gamma glutamyl transferase (GGT), serum potassium, white blood cells (WBC) and blood platelet (PLT) (*P* < 0.001). Conversely, higher scores were associated with lower HDL-C (*P* < 0.001). Figure 2 shows bar graphs of the relationship between quartiles of the four indexes and mortality. It is easy to see that both all-cause mortality and cardiovascular mortality broadly show a gradual increase as the four indexes rise [TyG index: all-cause mortality (5.16% vs. 8.70% vs. 11.63% vs. 15.21%, *P* < 0.001) and cardiovascular mortality (1.12% vs. 2.41% vs. 2.56% vs. 4.30%, *P* < 0.001); METS-IR: all-cause mortality (8.34% vs. 10.39% vs. 10.89% vs. 11.33%, *P* < 0.001) and cardiovascular mortality (1.74% vs. 2.76% vs. 2.37% vs. 3.58%, *P* < 0.001); TG/HDL-C: all-cause mortality (6.85% vs. 9.00% vs. 11.79% vs. 13.01%, *P* < 0.001) and cardiovascular mortality (1.62% vs. 2.35% vs. 2.82% vs. 3.56%, *P* < 0.001); HOMA-IR: all-cause mortality (8.31% vs. 9.68% vs. 10.36% vs. 13.32%, *P* < 0.001) and cardiovascular mortality (1.86% vs. 2.50% vs. 2.45% vs. 3.94%, *P* < 0.001)]. Comparison of baseline characteristics between the survival group and death group is shown in Table 3, with statistical differences in all variables except BMI (*P* < 0.05).

**Table 1:** Baseline characteristics according to TyG Index, METS-IR, TG/HDL-C and HOMA-IR quartiles, NHANES 2001–2018

	Overall		TyG index levels					METS-IR levels					TG/HDL-C levels					HOMA-IR levels				
Characteristic	N	Overall, N = 14,653	Q1, N = 3325 (22.69%)	Q2, N = 3658 (24.96%)	Q3, N = 3824 (26.10%)	Q4, N = 3846 (26.25%)	*P* Value	Q1, N = 3416 (23.31%)	Q2, N = 3719 (25.38%)	Q3, N = 3764 (25.69%)	Q4, N = 3754 (25.62%)	*P* Value	Q1, N = 3417 (23.32%)	Q2, N = 3684 (25.14%)	Q3, N = 3668 (25.03%)	Q4, N = 3884 (26.51%)	*P* Value	Q1, N = 3810 (26.00%)	Q2, N = 3643 (24.86%)	Q3, N = 3714 (25.35%)	Q4, N = 3486 (23.79%)	*P* Value
TyG index	14,653	8.63 (0.66)	7.83 (0.26)	8.37 (0.12)	8.79 (0.13)	9.49 (0.46)	** < 0.001**	8.15 (0.47)	8.47 (0.50)	8.78 (0.55)	9.11 (0.69)	** < 0.001**	7.90 (0.34)	8.38 (0.27)	8.76 (0.29)	9.40 (0.51)	** < 0.001**	8.23 (0.53)	8.51 (0.54)	8.79 (0.57)	9.12 (0.68)	** < 0.001**
METS-IR	14,653	42.92 (12.54)	34.87 (9.14)	39.51 (9.97)	44.75 (11.28)	52.26 (12.40)	** < 0.001**	29.41 (3.07)	37.43 (2.06)	45.13 (2.48)	60.00 (9.81)	** < 0.001**	33.77 (8.48)	39.50 (9.63)	45.11 (11.08)	52.44 (12.12)	** < 0.001**	33.63 (6.98)	39.51 (8.28)	45.90 (9.69)	56.11 (13.00)	** < 0.001**
TG/HDL-C	14,653	2.97 (3.83)	0.95 (0.35)	1.68 (0.52)	2.72 (0.89)	6.49 (6.31)	** < 0.001**	1.31 (0.78)	2.11 (1.38)	3.23 (2.62)	5.24 (6.38)	** < 0.001**	0.86 (0.23)	1.59 (0.23)	2.60 (0.38)	6.55 (6.05)	** < 0.001**	1.77 (2.74)	2.44 (2.89)	3.45 (3.80)	4.66 (5.17)	** < 0.001**
HOMA-IR	14,653	3.39 (5.23)	1.76 (1.47)	2.46 (2.99)	3.46 (3.43)	5.83 (8.79)	** < 0.001**	1.44 (1.19)	2.16 (1.55)	3.30 (2.54)	6.69 (9.10)	** < 0.001**	1.86 (3.01)	2.60 (3.15)	3.56 (3.99)	5.36 (8.05)	** < 0.001**	1.00 (0.32)	1.92 (0.28)	3.25 (0.53)	8.54 (9.57)	** < 0.001**
Age, years	14,653	46 (16.61)	40 (15.84)	46 (16.70)	48 (16.70)	51 (15.20)	** < 0.001**	43 (17.41)	47 (17.13)	48 (16.19)	47 (15.09)	** < 0.001**	44 (17.00)	46 (17.02)	48 (16.88)	48 (15.31)	** < 0.001**	44 (16.26)	46 (16.88)	47 (16.79)	49 (16.06)	** < 0.001**
Gender, n (%)	14,653						** < 0.001**					** < 0.001**					** < 0.001**					** < 0.001**
Male		7423 (49.82)	1340 (38.67)	1818 (47.97)	2034 (53.26)	2231 (58.84)		1345 (35.78)	2012 (53.39)	2128 (56.79)	1938 (53.70)		1246 (34.47)	1751 (45.53)	1951 (53.43)	2475 (64.45)		1851 (45.41)	1788 (47.50)	1956 (53.33)	1828 (54.47)	
Female		7230 (50.18)	1985 (61.33)	1840 (52.03)	1790 (46.74)	1615 (41.16)		2071 (64.22)	1707 (46.61)	1636 (43.21)	1816 (46.30)		2171 (65.53)	1933 (54.47)	1717 (46.57)	1409 (35.55)		1959 (54.59)	1855 (52.50)	1758 (46.67)	1658 (45.53)	
BMI, kg/m^2^	14,653						** < 0.001**					** < 0.001**					** < 0.001**					** < 0.001**
Underweight (< 18.5)		216 (1.53)	108 (3.41)	67 (1.83)	30 (0.72)	11 (0.23)		216 (6.00)	0 (0.00)	0 (0.00)	0 (0.00)		126 (3.90)	53 (1.47)	25 (0.65)	12 (0.25)		156 (3.92)	37 (1.08)	17 (0.39)	6 (0.15)	
Normal (18.5 to < 25)		4142 (29.76)	1537 (49.47)	1226 (35.02)	869 (23.11)	510 (12.33)		2955 (86.85)	1106 (29.03)	79 (1.57)	2 (0.01)		1597 (49.64)	1220 (35.90)	815 (22.83)	510 (12.31)		2185 (58.85)	1208 (32.72)	574 (14.65)	175 (4.37)	
Overweight (25 to < 30)		5006 (33.55)	967 (28.19)	1282 (35.45)	1361 (35.10)	1396 (35.10)		244 (7.14)	2457 (67.10)	2038 (54.44)	267 (6.34)		1003 (29.27)	1259 (33.23)	1281 (34.91)	1463 (36.44)		1151 (30.12)	1549 (42.40)	1475 (38.84)	831 (21.39)	
Obesity (30 or greater)		5289 (35.16)	713 (18.93)	1083 (27.70)	1564 (41.07)	1929 (52.34)		1 (0.01)	156 (3.87)	1647 (43.99)	3485 (93.65)		691 (17.19)	1152 (29.40)	1547 (41.61)	1899 (51.00)		318 (7.11)	849 (23.80)	1648 (46.12)	2474 (74.09)	
Race, n (%)	14,653						** < 0.001**					** < 0.001**					** < 0.001**					** < 0.001**
Mexican American		2496 (7.78)	379 (5.95)	547 (7.06)	729 (8.77)	841 (9.25)		361 (4.93)	614 (7.37)	790 (9.88)	731 (9.03)		367 (5.58)	577 (7.18)	704 (8.82)	848 (9.37)		440 (5.11)	575 (7.02)	729 (9.53)	752 (10.27)	
Other Hispanic		1172 (4.65)	226 (4.31)	281 (4.43)	334 (5.07)	331 (4.76)		223 (3.84)	310 (4.78)	341 (5.53)	298 (4.48)		227 (3.91)	284 (4.44)	322 (5.11)	339 (5.11)		242 (3.72)	297 (4.93)	323 (5.02)	310 (5.14)	
Non-Hispanic White		7058 (70.84)	1402 (65.87)	1791 (71.70)	1892 (71.61)	1973 (73.92)		1786 (73.18)	1790 (70.51)	1701 (69.41)	1781 (70.19)		1470 (66.57)	1785 (71.64)	1752 (70.46)	2051 (74.25)		2049 (74.82)	1824 (71.95)	1688 (67.92)	1497 (67.51)	
Non-Hispanic Black		2756 (10.54)	1000 (17.20)	761 (11.28)	559 (7.78)	436 (6.26)		573 (9.03)	682 (10.50)	709 (10.44)	792 (12.24)		1027 (17.09)	757 (11.10)	590 (9.02)	382 (5.52)		696 (9.61)	660 (10.16)	678 (10.33)	722 (12.53)	
Other/multiracial		1171 (6.19)	318 (6.67)	278 (5.53)	310 (6.77)	265 (5.81)		473 (9.02)	323 (6.84)	223 (4.74)	152 (4.06)		326 (6.85)	281 (5.64)	300 (6.59)	264 (5.75)		383 (6.74)	287 (5.94)	296 (7.20)	205 (4.55)	
PIR, n (%)	14,653						** < 0.001**					** < 0.001**					** < 0.001**					** < 0.001**
< 1.0		2879 (13.50)	646 (13.98)	702 (13.22)	749 (13.52)	782 (13.32)		652 (13.64)	667 (11.78)	721 (13.30)	839 (15.28)		635 (13.03)	714 (13.34)	693 (13.17)	837 (14.40)		681 (11.86)	678 (12.99)	752 (14.20)	768 (15.53)	
1.0–3.0		6121 (36.23)	1302 (33.61)	1466 (34.38)	1640 (38.80)	1713 (38.00)		1289 (32.61)	1570 (36.61)	1605 (36.48)	1657 (39.31)		1321 (32.97)	1522 (35.80)	1590 (37.94)	1688 (38.00)		1503 (33.71)	1504 (35.57)	1555 (35.91)	1559 (40.82)	
> 3.0		5653 (50.27)	1377 (52.41)	1490 (52.40)	1435 (47.68)	1351 (48.68)		1475 (53.75)	1482 (51.61)	1438 (50.22)	1258 (45.41)		1461 (54.00)	1448 (50.86)	1385 (48.89)	1359 (47.60)		1626 (54.43)	1461 (51.44)	1407 (49.89)	1159 (43.65)	
Education, n (%)	14,653						** < 0.001**					** < 0.001**					** < 0.001**					** < 0.001**
Below high school		1528 (5.38)	198 (3.31)	332 (4.85)	433 (5.97)	565 (7.29)		217 (3.39)	381 (5.11)	512 (7.09)	418 (6.01)		209 (3.39)	371 (4.94)	396 (5.98)	552 (7.05)		308 (4.18)	332 (4.53)	438 (6.22)	450 (7.04)	
High school		5558 (34.78)	1127 (29.18)	1363 (34.32)	1519 (37.09)	1549 (38.23)		1173 (30.31)	1390 (34.35)	1463 (36.96)	1532 (37.60)		1151 (29.06)	1355 (33.64)	1494 (37.57)	1558 (38.44)		1318 (30.77)	1406 (35.98)	1410 (35.05)	1424 (38.45)	
Above high school		7657 (59.84)	2000 (67.51)	1963 (60.83)	1872 (56.94)	1732 (54.48)		2026 (66.30)	1948 (60.54)	1789 (55.95)	1804 (56.39)		2057 (67.55)	1958 (61.42)	1778 (56.45)	1774 (54.51)		2184 (65.05)	1905 (59.49)	1866 (58.73)	1612 (54.51)	
Drinking, n (%)	14,653						** < 0.001**					** < 0.001**					** < 0.001**					** < 0.001**
Never		2060 (11.46)	515 (12.99)	495 (11.40)	494 (9.73)	556 (11.84)		514 (11.73)	475 (10.64)	537 (11.51)	534 (11.95)		522 (12.60)	525 (11.73)	525 (11.29)	488 (10.32)		468 (9.60)	506 (13.89)	527 (11.61)	559 (13.28)	
Former		3923 (24.63)	813 (21.28)	974 (24.23)	1086 (26.37)	1050 (26.46)		760 (19.73)	936 (22.18)	1007 (25.13)	1220 (31.58)		815 (21.09)	960 (23.36)	1057 (26.80)	1091 (27.05)		869 (20.48)	939 (25.78)	1003 (25.02)	1112 (31.63)	
Current		8670 (63.91)	1997 (65.73)	2189 (64.37)	2244 (63.90)	2240 (61.70)		2142 (68.54)	2308 (67.18)	2220 (63.36)	2000 (56.47)		2080 (66.31)	2199 (64.91)	2086 (61.91)	2305 (62.63)		2473 (69.92)	2198 (60.33)	2184 (63.37)	1815 (55.09)	
Smoking, n (%)	14,653						** < 0.001**					** < 0.001**					** < 0.001**					** < 0.001**
Never		7867 (53.25)	2126 (63.56)	1986 (53.58)	1976 (50.88)	1779 (45.46)		1944 (55.89)	1993 (52.64)	2005 (52.91)	1925 (51.49)		2145 (62.18)	2076 (55.67)	1876 (50.23)	1770 (45.64)		1985 (51.98)	2039 (55.94)	2008 (53.15)	1835 (51.85)	
Former		3400 (22.70)	563 (16.51)	802 (21.80)	947 (24.78)	1088 (27.42)		599 (17.67)	871 (22.83)	966 (25.04)	964 (25.40)		659 (19.54)	807 (21.51)	905 (24.49)	1029 (25.05)		756 (19.37)	789 (21.22)	927 (24.50)	928 (26.89)	
Current		3386 (24.05)	636 (19.93)	870 (24.62)	901 (24.34)	979 (27.12)		873 (26.44)	855 (24.53)	793 (22.05)	865 (23.11)		613 (18.28)	801 (22.82)	887 (25.28)	1085 (29.31)		1069 (28.65)	815 (22.84)	779 (22.35)	723 (21.26)	
SBP, mmHg	14,356	121 (17)	116 (16)	120 (17)	123 (16)	127 (17)	** < 0.001**	117 (17)	121 (17)	123 (16)	125 (16)	** < 0.001**	118 (17)	120 (17)	122 (17)	125 (17)	** < 0.001**	117 (17)	121 (17)	123 (16)	126 (16)	** < 0.001**
DBP, mmHg	14,356	70 (12)	67 (11)	69 (12)	71 (12)	73 (13)	** < 0.001**	68 (11)	68 (12)	71 (12)	73 (13)	** < 0.001**	68 (12)	69 (12)	70 (12)	73 (12)	** < 0.001**	68 (11)	69 (12)	71 (12)	72 (13)	** < 0.001**
Hypertension, n (%)	14,444						** < 0.001**					** < 0.001**					** < 0.001**					** < 0.001**
Non-hypertension		8315 (62.69)	2413 (79.31)	2224 (67.21)	2011 (57.95)	1667 (47.05)		2491 (78.79)	2268 (66.90)	1998 (59.45)	1558 (45.30)		2276 (73.46)	2182 (66.45)	1932 (59.32)	1925 (52.45)		2633 (75.95)	2288 (68.83)	1973 (57.28)	1421 (43.70)	
Hypertension		6129 (37.31)	859 (20.69)	1379 (32.79)	1759 (42.05)	2132 (52.95)		874 (21.21)	1389 (33.10)	1717 (40.55)	2149 (54.70)		1093 (26.54)	1454 (33.55)	1671 (40.68)	1911 (47.55)		1116 (24.05)	1299 (31.17)	1692 (42.72)	2022 (56.30)	
Diabetes, n (%)	14,613						** < 0.001**					** < 0.001**					** < 0.001**					** < 0.001**
Non-diabetes		11,863 (85.87)	3149 (96.37)	3271 (92.87)	3141 (86.97)	2302 (67.38)		3207 (96.14)	3252 (91.06)	2970 (85.23)	2434 (70.80)		3062 (92.74)	3140 (89.77)	2887 (84.35)	2774 (77.28)		3552 (95.60)	3270 (93.51)	3064 (86.56)	1977 (62.72)	
Diabetes		2750 (14.13)	171 (3.63)	379 (7.13)	672 (13.03)	1528 (32.62)		205 (3.86)	457 (8.94)	785 (14.77)	1303 (29.20)		349 (7.26)	540 (10.23)	766 (15.65)	1095 (22.72)		248 (4.40)	362 (6.49)	640 (13.44)	1500 (37.28)	
Hyperlipidemia, n (%)	14,653						** < 0.001**					** < 0.001**					** < 0.001**					** < 0.001**
Non-hyperlipidemia		3665 (26.31)	1832 (56.98)	1170 (33.22)	599 (15.21)	64 (1.26)		1621 (49.04)	1033 (28.24)	616 (16.94)	395 (10.43)		1787 (54.13)	1256 (35.66)	607 (17.38)	15 (0.43)		1530 (42.02)	1032 (28.50)	699 (18.30)	404 (11.72)	
Hyperlipidemia		10,988 (73.69)	1493 (43.02)	2488 (66.78)	3225 (84.79)	3782 (98.74)		1795 (50.96)	2686 (71.76)	3148 (83.06)	3359 (89.57)		1630 (45.87)	2428 (64.34)	3061 (82.62)	3869 (99.57)		2280 (57.98)	2611 (71.50)	3015 (81.70)	3082 (88.28)	
CVD, n (%)	14,458						** < 0.001**					** < 0.001**					** < 0.001**					** < 0.001**
Non-CVD		12,905 (91.58)	3005 (93.67)	3286 (90.85)	3357 (88.46)	3257 (84.86)		3092 (93.36)	3311 (90.00)	3322 (88.80)	3180 (85.35)		3075 (92.54)	3283 (90.47)	3213 (88.24)	3334 (86.26)		3466 (92.38)	3269 (91.16)	3265 (89.13)	2905 (84.03)	
CVD		1553 (8.42)	203 (6.33)	331 (9.15)	438 (11.54)	581 (15.14)		220 (6.64)	368 (10.00)	419 (11.20)	546 (14.65)		248 (7.46)	346 (9.53)	428 (11.76)	531 (13.74)		286 (7.62)	317 (8.84)	398 (10.87)	552 (15.97)	
MetS, n (%)	14,653						** < 0.001**					** < 0.001**					** < 0.001**					** < 0.001**
Non-MetS		8095 (58.77)	2875 (86.47)	2613 (71.43)	1938 (50.68)	669 (17.39)		3104 (90.87)	2560 (68.84)	1619 (43.01)	812 (21.63)		2790 (81.65)	2573 (69.84)	1905 (51.94)	827 (21.29)		3242 (85.09)	2436 (66.87)	1671 (44.99)	746 (21.40)	
MetS		6558 (41.23)	450 (13.53)	1045 (28.57)	1886 (49.32)	3177 (82.61)		312 (9.13)	1159 (31.16)	2145 (56.99)	2942 (78.37)		627 (18.35)	1111 (30.16)	1763 (48.06)	3057 (78.71)		568 (14.91)	1207 (33.13)	2043 (55.01)	2740 (78.60)	
All-cause mortality, n (%)	14,653	2085 (10.23)	249 (5.16)	479 (8.70)	611 (11.63)	746 (15.21)	** < 0.001**	431 (8.34)	551 (10.39)	561 (10.89)	542 (11.33)	** < 0.001**	337 (6.85)	499 (9.00)	592 (11.79)	657 (13.01)	** < 0.001**	504 (8.31)	487 (9.68)	513 (10.36)	581 (13.32)	** < 0.001**
Cardiovascular mortality, n (%)	14,653	549 (2.61)	57 (1.12)	129 (2.41)	147 (2.56)	216 (4.30)	** < 0.001**	86 (1.74)	148 (2.76)	146 (2.37)	169 (3.58)	** < 0.001**	86 (1.62)	119 (2.35)	155 (2.82)	189 (3.56)	** < 0.001**	127 (1.86)	126 (2.50)	124 (2.45)	172 (3.94)	** < 0.001**

The data was shown as mean (SD) for continuous, n (%) for categorical

*TyG index* triglyceride glucose index, *METS-IR* metabolic score for Insulin resistance, *TG* triglyceride, *HDL-C* high-density lipoprotein-cholesterol, *HOMA-IR* homeostatic model assessment of insulin resistance, *BMI* body mass index, *PIR* the ratio of family income to poverty, *SBP* systolic pressure, *DBP* diastolic pressure, *CVD* cardiovascular disease, *MetS* metabolic syndrome

*P* values in bold meant significantly different (*P* < 0.05)

**Table 2:** Baseline levels of laboratory characteristics according to TyG Index, METS-IR, TG/HDL-C and HOMA-IR quartiles, NHANES 2001–2018

Overall			TyG index levels					METS-IR levels					TG/HDL-C levels					HOMA-IR levels				
Characteristic	N	Overall, N = 14,653	Q1, N = 3325 (22.69%)	Q2, N = 3658 (24.96%)	Q3, N = 3824 (26.10%)	Q4, N = 3846 (26.25%)	*P* Value	Q1, N = 3416 (23.31%)	Q2, N = 3719 (25.38%)	Q3, N = 3764 (25.69%)	Q4, N = 3754 (25.62%)	*P* Value	Q1, N = 3417 (23.32%)	Q2, N = 3684 (25.14%)	Q3, N = 3668 (25.03%)	Q4, N = 3884 (26.51%)	*P* Value	Q1, N = 3810 (26.00%)	Q2, N = 3643 (24.86%)	Q3, N = 3714 (25.35%)	Q4, N = 3486 (23.79%)	*P* Value
HbA1C, %	14,622	5.57 (0.90)	5.27 (0.43)	5.40 (0.49)	5.53 (0.65)	6.06 (1.43)	** < 0.001**	5.28 (0.45)	5.43 (0.66)	5.59 (0.87)	5.97 (1.27)	** < 0.001**	5.36 (0.57)	5.47 (0.75)	5.61 (0.90)	5.80 (1.18)	** < 0.001**	5.30 (0.52)	5.38 (0.56)	5.56 (0.75)	6.15 (1.40)	** < 0.001**
FINS, pmol/L	14,653	73.43 (80.65)	45.30 (37.02)	59.12 (50.71)	79.26 (62.74)	109.22 (126.05)	** < 0.001**	36.42 (24.52)	52.01 (33.99)	75.19 (49.25)	130.85 (129.30)	** < 0.001**	44.50 (42.59)	59.98 (50.90)	78.74 (69.16)	107.68 (118.35)	** < 0.001**	26.29 (8.34)	47.89 (7.96)	76.92 (15.23)	163.96 (135.17)	** < 0.001**
FPG, mmol/L	14,653	5.80 (1.61)	5.15 (0.54)	5.46 (0.70)	5.74 (0.97)	6.83 (2.66)	** < 0.001**	5.23 (0.72)	5.54 (0.97)	5.87 (1.47)	6.57 (2.40)	** < 0.001**	5.38 (0.97)	5.59 (1.20)	5.85 (1.52)	6.32 (2.23)	** < 0.001**	5.17 (0.71)	5.47 (0.79)	5.82 (1.17)	7.01 (2.66)	** < 0.001**
LDL-C, mmol/L	14,343	2.98 (0.91)	2.60 (0.74)	2.99 (0.83)	3.18 (0.91)	3.15 (1.01)	** < 0.001**	2.75 (0.85)	3.03 (0.91)	3.16 (0.92)	3.00 (0.90)	** < 0.001**	2.66 (0.78)	2.97 (0.85)	3.12 (0.91)	3.17 (0.99)	** < 0.001**	2.86 (0.88)	3.05 (0.88)	3.08 (0.94)	2.96 (0.91)	** < 0.001**
HDL-C, mmol/L	14,653	1.39 (0.42)	1.64 (0.45)	1.47 (0.39)	1.32 (0.34)	1.13 (0.29)	** < 0.001**	1.74 (0.44)	1.44 (0.34)	1.25 (0.28)	1.12 (0.28)	** < 0.001**	1.81 (0.43)	1.47 (0.29)	1.26 (0.25)	1.04 (0.22)	** < 0.001**	1.60 (0.44)	1.43 (0.40)	1.30 (0.37)	1.16 (0.29)	** < 0.001**
TC, mmol/L	14,653	5.04 (1.08)	4.54 (0.88)	4.92 (0.94)	5.18 (1.00)	5.51 (1.22)	** < 0.001**	4.92 (1.00)	5.03 (1.05)	5.15 (1.10)	5.07 (1.14)	** < 0.001**	4.78 (0.95)	4.91 (0.99)	5.04 (1.03)	5.41 (1.21)	** < 0.001**	4.94 (1.03)	5.09 (1.05)	5.14 (1.12)	5.02 (1.11)	** < 0.001**
TG, mmol/L	14,653	1.50 (1.35)	0.63 (0.15)	1.01 (0.15)	1.47 (0.25)	2.86 (2.11)	** < 0.001**	0.93 (0.46)	1.23 (0.66)	1.62 (1.04)	2.22 (2.16)	** < 0.001**	0.66 (0.19)	1.01 (0.22)	1.42 (0.31)	2.79 (2.07)	** < 0.001**	1.06 (0.88)	1.33 (1.05)	1.69 (1.40)	2.05 (1.83)	** < 0.001**
Scr, umol/L	14,638	78.71 (34.06)	75.07 (34.42)	78.36 (30.96)	80.10 (33.53)	81.14 (36.90)	** < 0.001**	75.38 (36.43)	80.28 (37.93)	79.85 (26.35)	79.42 (33.93)	** < 0.001**	75.05 (33.07)	77.62 (33.58)	79.89 (32.78)	81.99 (36.12)	** < 0.001**	78.55 (42.81)	78.83 (36.97)	78.62 (26.55)	78.90 (23.01)	**0.011**
Uric acid, umol/L	14,638	327.62 (82.37)	290.17 (72.04)	316.37 (75.90)	341.18 (79.50)	361.19 (84.08)	** < 0.001**	283.23 (69.26)	318.32 (74.40)	342.25 (77.56)	367.79 (83.07)	** < 0.001**	288.44 (70.76)	315.05 (74.99)	338.11 (80.52)	365.48 (82.04)	** < 0.001**	298.61 (73.66)	317.57 (76.08)	340.39 (80.29)	364.17 (86.23)	** < 0.001**
BUN, mmol/L	14,639	4.72 (1.86)	4.48 (1.64)	4.65 (1.78)	4.76 (1.92)	4.97 (2.05)	** < 0.001**	4.45 (1.74)	4.77 (1.80)	4.78 (1.81)	4.87 (2.07)	** < 0.001**	4.61 (1.73)	4.66 (1.79)	4.74 (1.94)	4.84 (1.97)	** < 0.001**	4.54 (1.79)	4.69 (1.86)	4.76 (1.73)	4.93 (2.08)	** < 0.001**
Albumin, g/L	14,639	42.66 (3.17)	43.02 (3.18)	42.66 (3.16)	42.51 (3.15)	42.48 (3.17)	** < 0.001**	43.55 (3.15)	43.02 (3.00)	42.58 (2.92)	41.48 (3.24)	** < 0.001**	42.95 (3.11)	42.73 (3.22)	42.38 (3.24)	42.60 (3.10)	** < 0.001**	43.16 (3.18)	42.91 (3.07)	42.65 (3.02)	41.70 (3.26)	** < 0.001**
LDH, IU/L	14,619	127.49 (28.92)	124.76 (28.15)	127.81 (32.93)	128.30 (26.44)	128.96 (27.47)	** < 0.001**	123.02 (26.53)	126.34 (25.68)	128.56 (33.78)	132.17 (28.42)	** < 0.001**	127.09 (27.25)	126.65 (26.85)	127.80 (33.43)	128.41 (27.83)	0.12	126.06 (29.73)	126.23 (26.16)	127.45 (32.45)	131.00 (26.22)	** < 0.001**
ALT, IU/L	14,623	25.88 (26.36)	21.62 (22.87)	23.62 (15.34)	26.84 (17.12)	31.34 (41.24)	** < 0.001**	20.87 (22.26)	24.60 (38.84)	27.54 (18.68)	30.65 (19.15)	** < 0.001**	21.48 (22.38)	23.19 (15.83)	26.69 (16.59)	31.73 (40.39)	** < 0.001**	21.84 (20.60)	23.25 (15.71)	27.58 (40.29)	32.54 (20.98)	** < 0.001**
AST, IU/L	14,621	25.39 (17.80)	24.26 (22.97)	24.61 (18.88)	25.43 (12.74)	27.23 (15.11)	** < 0.001**	24.34 (19.65)	24.81 (12.50)	26.04 (21.98)	26.39 (15.54)	** < 0.001**	24.50 (20.45)	24.31 (16.61)	25.39 (16.24)	27.23 (17.58)	** < 0.001**	24.86 (19.58)	24.00 (13.08)	25.27 (19.75)	27.90 (17.51)	** < 0.001**
TBil, umol/L	14,633	12.74 (5.16)	12.70 (5.30)	13.01 (5.40)	12.71 (5.10)	12.54 (4.79)	0.15	13.22 (5.26)	13.15 (5.36)	12.78 (5.02)	11.82 (4.86)	** < 0.001**	12.72 (5.25)	12.87 (5.22)	12.73 (5.30)	12.65 (4.87)	0.80	13.47 (5.47)	13.03 (5.14)	12.36 (4.85)	11.86 (4.90)	** < 0.001**
GGT, IU/L	14,639	28.21 (39.76)	19.73 (23.78)	23.34 (23.67)	28.81 (31.76)	40.78 (62.89)	** < 0.001**	20.87 (28.32)	25.75 (30.61)	30.08 (40.94)	36.31 (52.87)	** < 0.001**	21.47 (31.50)	23.46 (24.23)	28.90 (31.87)	38.27 (58.63)	** < 0.001**	22.21 (29.44)	23.96 (27.02)	29.73 (32.49)	39.68 (63.08)	** < 0.001**
Serum K, mmol/L	14,638	4.03 (0.33)	4.00 (0.32)	4.03 (0.33)	4.04 (0.34)	4.07 (0.34)	** < 0.001**	4.01 (0.34)	4.03 (0.34)	4.04 (0.32)	4.06 (0.32)	** < 0.001**	4.01 (0.33)	4.03 (0.33)	4.04 (0.34)	4.06 (0.33)	** < 0.001**	4.02 (0.34)	4.02 (0.33)	4.05 (0.32)	4.06 (0.34)	** < 0.001**
Serum Na, mmol/L	14,639	139.13 (2.16)	139.23 (2.16)	139.26 (2.07)	139.21 (2.08)	138.83 (2.29)	** < 0.001**	139.14 (2.19)	139.33 (2.23)	139.19 (2.05)	138.87 (2.14)	** < 0.001**	139.22 (2.24)	139.26 (2.05)	139.15 (2.16)	138.90 (2.18)	** < 0.001**	139.17 (2.19)	139.26 (2.10)	139.16 (2.07)	138.88 (2.27)	** < 0.001**
Serum Fe, umol/L	14,631	16.22 (6.54)	15.68 (6.90)	16.39 (6.81)	16.37 (6.30)	16.38 (6.12)	** < 0.001**	17.33 (7.28)	16.61 (6.63)	16.26 (6.17)	14.65 (5.66)	** < 0.001**	16.00 (6.95)	16.41 (6.72)	16.13 (6.47)	16.29 (6.04)	0.06	17.21 (7.20)	16.61 (6.58)	15.88 (6.07)	14.78 (5.78)	** < 0.001**
WBC, *10^9/L	14,627	6.78 (2.19)	6.13 (1.81)	6.65 (2.36)	6.95 (2.21)	7.35 (2.14)	** < 0.001**	6.29 (1.95)	6.57 (2.45)	6.84 (2.00)	7.42 (2.17)	** < 0.001**	6.09 (1.87)	6.58 (2.08)	7.03 (2.23)	7.35 (2.32)	** < 0.001**	6.28 (2.34)	6.62 (2.18)	6.94 (2.00)	7.45 (2.01)	** < 0.001**
PLT, *10^9/L	14,626	251.13 (66.10)	242.04 (62.14)	251.31 (65.92)	254.86 (66.94)	255.81 (68.22)	** < 0.001**	247.52 (62.97)	246.04 (64.22)	251.26 (64.76)	259.79 (71.34)	** < 0.001**	241.84 (61.92)	251.14 (65.30)	256.04 (68.23)	254.96 (67.67)	** < 0.001**	246.55 (64.40)	250.52 (64.12)	253.34 (64.61)	255.51 (71.84)	** < 0.001**
Hemoglobin, g/L	14,627	144.44 (14.69)	139.98 (14.17)	143.55 (14.57)	145.81 (14.44)	148.23 (14.33)	** < 0.001**	141.35 (13.63)	145.05 (14.46)	146.01 (14.66)	145.45 (15.52)	** < 0.001**	139.34 (13.83)	143.33 (14.32)	145.26 (14.72)	149.37 (14.08)	** < 0.001**	142.43 (14.20)	144.52 (14.31)	145.68 (15.05)	145.64 (15.08)	** < 0.001**

The data was shown as mean (SD)

*TyG index* triglyceride glucose index, *METS-IR* metabolic score for Insulin resistance, *TG* triglyceride, *HDL-C* high-density lipoprotein-cholesterol, *HOMA-IR* homeostatic model assessment of insulin resistance, *HbA1C* glycosylated hemoglobin, *FINS* fasting serum insulin, *FPG* fasting plasma glucose, *LDL-C* low-density lipoprotein-cholesterol, *TC* cholesterol, *Scr* serum creatinine, *BUN* blood urea nitrogen, *LDH* lactate dehydrogenase, *ALT* alanine transaminase, *AST* aspartate transaminase, *TBil* total bilirubin, *GGT* gamma glutamyl transferase, *K* potassium, *Na* sodium, *Fe* iron, *WBC* white blood cells, *PLT* blood platelet

*P* values in bold meant significantly different (*P* < 0.05)

**Table 3:** Baseline characteristics according to different prognoses, NHANES 2001–2018

Overall			All-cause mortality			Cardiovascular mortality		
Characteristic	N	Overall N = 14,653	Survival group N = 12,568 (89.77%)	Death group N = 2085 (10.23%)	*P* Value	Non-cardiovascular death group N = 14,104 (97.39%)	Cardiovascular death group N = 549 (2.61%)	*P* Value
Age, years	14,653	46 (17)	44 (15)	65 (14)	** < 0.001**	46 (16)	66 (14)	** < 0.001**
Gender, n (%)	14,653				** < 0.001**			** < 0.001**
Male		7423 (49.82)	6199 (49.24)	1224 (54.88)		7080 (49.57)	343 (59.06)	
Female		7230 (50.18)	6369 (50.76)	861 (45.12)		7024 (50.43)	206 (40.94)	
BMI, kg/m^2^	14,653				0.10			**0.009**
Underweight (< 18.5)		216 (1.53)	177 (1.46)	39 (2.09)		210 (1.54)	6 (1.15)	
Normal (18.5 to < 25)		4142 (29.76)	3556 (30.02)	586 (27.48)		4012 (29.92)	130 (24.06)	
Overweight (25 to < 30)		5006 (33.55)	4255 (33.44)	751 (34.52)		4813 (33.60)	193 (31.48)	
Obesity (30 or greater)		5289 (35.16)	4580 (35.08)	709 (35.91)		5069 (34.94)	220 (43.31)	
Race, n (%)	14,653				** < 0.001**			**0.002**
Mexican American		2496 (7.78)	2258 (8.25)	238 (3.67)		2432 (7.87)	64 (4.24)	
Other Hispanic		1172 (4.65)	1082 (4.85)	90 (2.91)		1153 (4.72)	19 (2.13)	
Non-Hispanic White		7058 (70.84)	5725 (69.90)	1333 (79.11)		6720 (70.68)	338 (76.71)	
Non-Hispanic Black		2756 (10.54)	2393 (10.54)	363 (10.61)		2643 (10.48)	113 (12.93)	
Other/multiracial		1171 (6.19)	1110 (6.46)	61 (3.70)		1156 (6.25)	15 (3.99)	
PIR, n (%)	14,653				** < 0.001**			** < 0.001**
< 1.0		2879 (13.50)	2479 (13.33)	400 (15.03)		2769 (13.47)	110 (14.79)	
1.0–3.0		6121 (36.23)	5048 (34.93)	1073 (47.65)		5840 (35.88)	281 (49.40)	
> 3.0		5653 (50.27)	5041 (51.74)	612 (37.32)		5495 (50.65)	158 (35.81)	
Education, n (%)	14,653				** < 0.001**			** < 0.001**
Below high school		1528 (5.38)	1163 (4.69)	365 (11.47)		1428 (5.21)	100 (11.92)	
High school		5558 (34.78)	4644 (33.69)	914 (44.32)		5318 (34.49)	240 (45.57)	
Above high school		7567 (59.84)	6761 (61.62)	806 (44.21)		7358 (60.30)	209 (42.51)	
Drinking, n (%)	14,653				**0.004**			**0.012**
Never		2060 (11.46)	1751 (11.21)	309 (13.61)		1972 (11.39)	88 (14.12)	
Former		3923 (24.63)	3355 (24.38)	568 (26.85)		3762 (24.51)	161 (29.11)	
Current		8670 (63.91)	7462 (64.41)	1208 (59.54)		8370 (64.10)	300 (56.77)	
Smoking, n (%)	14,653				** < 0.001**			** < 0.001**
Never		7867 (53.25)	7079 (55.15)	788 (36.57)		7631 (53.53)	236 (42.70)	
Former		3400 (22.70)	2596 (21.11)	804 (36.62)		3199 (22.41)	201 (33.51)	
Current		3386 (24.05)	2893 (23.74)	493 (26.81)		3274 (24.06)	112 (23.79)	
SBP, mmHg	14,356	121 (17)	120 (16)	132 (22)	** < 0.001**	121 (17)	134 (24)	** < 0.001**
DBP, mmHg	14,356	70 (12)	70 (12)	67 (16)	** < 0.001**	70 (12)	66 (17)	** < 0.001**
Hypertension, n (%)	14,444				** < 0.001**			** < 0.001**
Non-hypertension		8315 (62.69)	7738 (66.33)	577 (30.79)		8186 (63.70)	129 (24.74)	
Hypertension		6129 (37.31)	4641 (33.67)	1488 (69.21)		5714 (36.30)	415 (75.26)	
Diabetes, n (%)	14,613				** < 0.001**			** < 0.001**
Non-diabetes		11,863 (85.87)	10,537 (88.13)	1326 (66.04)		11,542 (86.60)	321 (58.78)	
Diabetes		2750 (14.13)	1997 (11.87)	753 (33.96)		2523 (13.40)	227 (41.22)	
Hyperlipidemia, n (%)	14,653				** < 0.001**			** < 0.001**
Non-hyperlipidemia		3665 (26.31)	3307 (27.43)	358 (16.49)		3591 (26.66)	74 (13.39)	
Hyperlipidemia		10,988 (73.69)	9261 (72.57)	1727 (83.51)		10,513 (73.34)	475 (86.61)	
CVD, n (%)	14,458				** < 0.001**			** < 0.001**
Non-CVD		12,905 (91.58)	11,475 (92.73)	1430 (68.62)		12,579 (90.44)	326 (59.38)	
CVD		1553 (8.42)	899 (7.27)	654 (31.38)		1330 (9.56)	223 (40.62)	
MetS, n (%)	14,653				** < 0.001**			** < 0.001**
Non-MetS		8095 (58.77)	7307 (58.14)	788 (37.79)		7913 (56.10)	182 (33.15)	
MetS		6558 (41.23)	5261 (41.86)	1297 (62.21)		6191 (43.90)	367 (66.85)	
TyG index	14,653	8.63 (0.66)	8.60 (0.66)	8.87 (0.66)	** < 0.001**	8.62 (0.66)	8.91 (0.69)	** < 0.001**
METS-IR	14,653	42.92 (12.54)	42.80 (12.51)	43.97 (12.74)	** < 0.001**	42.83 (12.49)	46.01 (13.71)	** < 0.001**
TG/HDL-C	14,653	2.97 (3.83)	2.92 (3.90)	3.33 (3.08)	** < 0.001**	2.95 (3.85)	3.47 (3.16)	** < 0.001**
HOMA-IR	14,653	3.39 (5.23)	3.29 (5.01)	4.24 (6.81)	** < 0.001**	3.35 (5.16)	4.68 (7.34)	** < 0.001**

The data was shown as mean (SD) for continuous, n (%) for categorical

*BMI* body mass index, *PIR* the ratio of family income to poverty, *SBP* systolic pressure, *DBP* diastolic pressure, *CVD* cardiovascular disease, *MetS* metabolic syndrome, *TyG index* triglyceride glucose index, *METS-IR* metabolic score for Insulin resistance, *TG* triglyceride, *HDL-C* high-density lipoprotein-cholesterol, *HOMA-IR* homeostatic model assessment of insulin resistance

*P* values in bold meant significantly different (*P* < 0.05)

Quartile 1: 5.65 ≤ TyG index ≤ 8.19; 18.80 ≤ METS-IR ≤ 34.32; 0.16 ≤ TG/HDL-C ≤ 1.26; 0.03 ≤ HOMA-IR ≤ 1.45.

Quartile 2: 8.19 < TyG index ≤ 8.61; 34.32 < METS-IR ≤ 41.43; 1.26 < TG/HDL-C ≤ 2.07; 1.45 < HOMA-IR ≤ 2.42.

Quartile 3: 8.61 < TyG index ≤ 9.05; 41.43 < METS-IR ≤ 49.95; 2.07 < TG/HDL-C ≤ 3.50; 2.42 < HOMA-IR ≤ 4.18.

Quartile 4: 9.05 < TyG index ≤ 13.40; 49.95 < METS-IR ≤ 193.33; 3.50 < TG/HDL-C ≤ 141.61; 4.18 < HOMA-IR ≤ 269.41.

### Feature selection

**Fig. 3:**
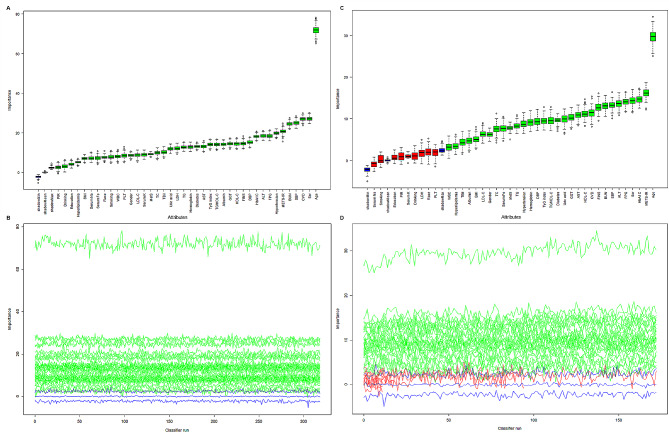
Feature selection process for all-cause mortality based on Boruta’s algorithm (**A**) and the value evolution of Z-score in the screening process (**B**). Feature selection process for cardiovascular mortality based on Boruta’s algorithm (**C**) and the value evolution of Z-score in the screening process (**D**). In **A** and **C**, the horizontal axis represents the variable name and the vertical axis represents the Z-values of each variable. In **B** and **D**, the horizontal axis represents the number of iterations, and the vertical axis represents the change in Z-values during the screening process. The blue boxes and lines correspond to the minimum, average, and maximum Z-scores for a shadow feature. The green boxes and lines represent the confirmed variables and the red ones represent the rejected variables in the model calculation. *CVD* cardiovascular disease, *Scr* serum creatinine, *SBP* systolic pressure, *BUN* blood urea nitrogen, *METS-IR* metabolic score for Insulin resistance, *FPG* fasting plasma glucose, *ALT* alanine transaminase, *HbA1C* glycosylated hemoglobin, *DBP* diastolic pressure, *HDL-C* high-density lipoprotein-cholesterol, *GGT* gamma glutamyl transferase, *HOMA-IR* homeostatic model assessment of insulin resistance, *TG* triglyceride, *TyG index* triglyceride glucose index, *FINS* fasting serum insulin, *AST* aspartate transaminase, *LDH* lactate dehydrogenase, *TBil* total bilirubin, *MetS* metabolic syndrome, *TC* cholesterol, *K* potassium, *LDL-C* low-density lipoprotein-cholesterol, *PLT* blood platelet, *WBC* white blood cells, *Fe* iron, *Na* sodium, *BMI* body mass index, *PIR* the ratio of family income to poverty

The results of feature screening based on Boruta’s algorithm are shown in Fig. 3. After 500 iterations it was determined that the six variables most closely associated with all-cause mortality (in order of z-value, excluding the insulin resistance replacement index) were age, CVD, Scr, SBP, BUN and hypertension, and the six variables most closely associated with cardiovascular mortality were age, HbA1C, FPG, Scr, ALT and SBP. Although several important characteristics such as gender, race, BMI, PIR, education, drinking and smoking were omitted due to low z-values compared with the most strongly associated or shaded characteristics, they were still included in the subsequent analyses on the basis of previous studies and clinical experience.

### Relationship between the IR replacement index and mortality

We fitted three Cox regression models to investigate the independent correlations between TyG index, METS-IR, TG/HDL-C and HOMA-IR levels and risk of mortality, respectively (Table 4). In conjunction with the feature selection outcomes of the Boruta algorithm, for all-cause mortality, the variables adjusted in Model 3 included age, gender, race, BMI, PIR, education, drinking, smoking, CVD, Scr, SBP, BUN, hypertension. For cardiovascular mortality, the adjusted variables in Model 3 were age, gender, race, BMI, PIR, education, drinking, smoking, HbA1C, FPG, Scr, ALT, SBP. The analysis shows that in Model 1 (unadjusted) and Model 2 (adjusted for age, gender, race), when treated as a continuous variable, the TyG index exhibits a significant correlation with both all-cause mortality and cardiovascular mortality [Model 1: all-cause mortality: HR (95% CI) 1.595 (1.484–1.714), *P* < 0.001; cardiovascular mortality: HR (95% CI) 1.702 (1.514–1.913), *P* < 0.001; Model 2: all-cause mortality: HR (95% CI) 1.248 (1.150–1.355), *P* < 0.001; cardiovascular mortality: HR (95% CI) 1.375 (1.173–1.612), *P* < 0.001]; similarly, METS-IR demonstrates a significant correlation with both all-cause mortality and cardiovascular mortality [Model 1: all-cause mortality: HR (95% CI) 1.008 (1.004–1.013), *P* < 0.001; cardiovascular mortality: HR (95% CI) 1.019 (1.012–1.026), *P* < 0.001; Model 2: all-cause mortality: HR (95% CI) 1.009 (1.004–1.014), *P* < 0.001; cardiovascular mortality: HR (95% CI) 1.024 (1.015–1.032), *P* < 0.001]; there was also a correlation between TG/HDL-C and all-cause and cardiovascular mortality [Model 1: all-cause mortality: HR (95% CI) 1.011 (1.002–1.019), *P* = 0.012; cardiovascular mortality: HR (95% CI) 1.015 (1.004–1.025), *P* = 0.008; Model 2: all-cause mortality: HR (95% CI) 1.013 (1.003–1.024), *P* = 0.009; cardiovascular mortality: HR (95% CI) 1.019 (1.005–1.033), *P* = 0.007]; finally, HOMA-IR was similarly significantly correlated with both all-cause and cardiovascular mortality [Model 1: all-cause mortality: HR (95% CI) 1.018 (1.012–1.023), *P* < 0.001; cardiovascular mortality: HR (95% CI) 1.019 (1.013–1.026), *P* < 0.001; Model 2: all-cause mortality: HR (95% CI) 1.016 (1.012–1.021), *P* < 0.001; cardiovascular mortality: HR (95% CI) 1.019 (1.014–1.025), *P* < 0.001]. In model 3, only METS-IR as a continuous variable was associated with both all-cause and cardiovascular mortality [all-cause mortality: HR (95% CI) 1.015 (1.008–1.022), *P* < 0.001; cardiovascular mortality: HR (95% CI) 1.018 (1.004–1.032), *P* = 0.012]. When considered as a categorical variable, all four indexes show a significant correlation with both all-cause mortality and cardiovascular mortality in the three Cox regression models (*P* < 0.001), although not all models demonstrate higher HR for patients with moderate and high scores.

**Table 4:** HRs (95% CIs) for mortality according to TyG Index, METS-IR, TG/HDL-C and HOMA-IR quartiles, NHANES 2001–2018

	All-cause mortality		Cardiovascular mortality	
Model	HR (95%CI)	*P* Value	HR (95%CI)	*P* Value
TyG index				
Model 1				
Continuous	1.595 (1.485–1.714)	< 0.001***	1.702 (1.514–1.913)	< 0.001***
Quartiles				
Q1	1		1	
Q2	1.571 (1.271–1.942)	< 0.001***	2.012 (1.290–3.140)	0.002**
Q3	2.090 (1.744–2.505)	< 0.001***	2.128 (1.422–3.185)	< 0.001***
Q4	2.711 (2.232–3.294)	< 0.001***	3.545 (2.462–5.104)	< 0.001***
*P* for trend		< 0.001***		< 0.001***
Model 2				
Continuous	1.248 (1.150–1.355)	< 0.001***	1.375 (1.173–1.612)	< 0.001***
Quartiles				
Q1	1		1	
Q2	0.959 (0.782–1.177)	0.690	1.208 (0.787–1.854)	0.387
Q3	1.022 (0.860–1.215)	0.810	1.029 (0.686–1.545)	0.889
Q4	1.218 (1.018–1.458)	0.031*	1.583 (1.093–2.292)	0.015*
*P* for trend		< 0.001***		< 0.001***
Model 3				
Continuous	1.130 (1.039–1.230)	0.004**	0.959 (0.765–1.201)	0.713
Quartiles				
Q1	1		1	
Q2	0.930 (0.757–1.142)	0.486	1.097 (0.715–1.681)	0.672
Q3	0.905 (0.752–1.089)	0.290	0.836 (0.547–1.278)	0.408
Q4	1.038 (0.863–1.247)	0.695	0.992 (0.660–1.492)	0.971
*P* for trend		< 0.001***		< 0.001***
METS-IR				
Model 1				
Continuous	1.008 (1.004–1.013)	< 0.001***	1.019 (1.012–1.026)	< 0.001***
Quartiles				
Q1	1		1	
Q2	1.251 (1.098–1.425)	< 0.001***	1.597 (1.146–2.226)	0.006**
Q3	1.315 (1.148–1.505)	< 0.001***	1.379 (0.995–1.910)	0.054
Q4	1.433 (1.246–1.648)	< 0.001***	2.181 (1.569–3.032)	< 0.001***
*P* for trend		< 0.001***		< 0.001***
Model 2				
Continuous	1.009 (1.004–1.014)	< 0.001***	1.024 (1.015–1.032)	< 0.001***
Quartiles				
Q1	1		1	
Q2	0.859 (0.758–0.974)	0.017*	1.046 (0.761–1.439)	0.781
Q3	0.890 (0.786–1.006)	0.063	0.887 (0.644–1.221)	0.462
Q4	1.157 (1.008–1.328)	0.038*	1.721 (1.240–2.390)	0.001**
*P* for trend		< 0.001***		< 0.001***
Model 3				
Continuous	1.015 (1.008–1.022)	< 0.001***	1.018 (1.004–1.032)	0.012*
Quartiles				
Q1	1		1	
Q2	0.931 (0.776–1.117)	0.441	0.957 (0.617–1.483)	0.843
Q3	1.034 (0.821–1.302)	0.778	0.643 (0.382–1.084)	0.098
Q4	1.222 (0.933–1.601)	0.146	0.868 (0.486–1.550)	0.631
*P* for trend		< 0.001***		< 0.001***
TG/HDL-C				
Model 1				
Continuous	1.011 (1.002–1.019)	0.012*	1.015 (1.004–1.025)	0.008**
Quartiles				
Q1	1		1	
Q2	1.197 (0.988–1.450)	0.067	1.319 (0.930–1.872)	0.121
Q3	1.551 (1.312–1.834)	< 0.001***	1.571 (1.135–2.174)	0.006**
Q4	1.659 (1.403–1.961)	< 0.001***	1.917 (1.399–2.627)	< 0.001***
*P* for trend		< 0.001***		< 0.001***
Model 2				
Continuous	1.013 (1.003–1.024)	0.009**	1.019 (1.005–1.033)	0.007**
Quartiles				
Q1	1		1	
Q2	1.021 (0.841–1.240)	0.834	1.117 (0.781–1.596)	0.544
Q3	1.143 (0.962–1.359)	0.130	1.145(0.821–1.598)	0.425
Q4	1.310 (1.116–1.539)	< 0.001***	1.510 (1.091–2.090)	0.013*
*P* for trend		< 0.001***		< 0.001***
Model 3				
Continuous	1.003 (0.991–1.014)	0.630	0.996 (0.972–1.021)	0.748
Quartiles				
Q1	1		1	
Q2	0.935 (0.765–1.144)	0.516	0.961 (0.666–1.387)	0.832
Q3	0.978 (0.807–1.184)	0.816	0.894 (0.625–1.280)	0.541
Q4	1.075 (0.917–1.259)	0.372	1.014 (0.714–1.441)	0.937
*P* for trend		< 0.001***		< 0.001***
HOMA-IR				
Model 1				
Continuous	1.018 (1.012–1.023)	< 0.001***	1.019 (1.013–1.026)	< 0.001***
Quartiles				
Q1	1		1	
Q2	1.170 (1.009–1.357)	0.037*	1.355 (0.995–1.844)	0.054
Q3	1.320 (1.128–1.544)	< 0.001***	1.400 (1.023–1.918)	0.036*
Q4	1.822 (1.555–2.135)	< 0.001***	2.424 (1.786–3.291)	< 0.001***
*P* for trend		< 0.001***		< 0.001***
Model 2				
Continuous	1.016 (1.012–1.021)	< 0.001***	1.019 (1.014–1.025)	< 0.001***
Quartiles				
Q1	1		1	
Q2	0.983 (0.857–1.128)	0.808	1.129 (0.834–1.529)	0.433
Q3	0.954 (0.821–1.110)	0.543	0.982 (0.715–1.349)	0.910
Q4	1.289 (1.117–1.488)	< 0.001***	1.677 (1.254–2.244)	< 0.001***
*P* for trend		< 0.001***		< 0.001***
Model 3				
Continuous	1.016 (1.011–1.021)	< 0.001***	1.008 (0.999–1.018)	0.071
Quartiles				
Q1	1		1	
Q2	1.042 (0.901–1.207)	0.578	1.132 (0.808–1.585)	0.471
Q3	0.984 (0.824–1.174)	0.856	0.880 (0.580–1.335)	0.548
Q4	1.254 (1.041–1.511)	0.017*	1.128 (0.736–1.729)	0.581
*P* for trend		< 0.001***		< 0.001***

Model 1: unadjusted

Model 2: adjusted for age, gender, race

Model 3 (All-cause mortality): adjusted for age, gender, race, BMI, PIR, education, drinking, smoking, CVD, Scr, SBP, BUN, hypertension

Model 3 (Cardiovascular mortality): adjusted for age, gender, race, BMI, PIR, education, drinking, smoking, HbA1C, FPG, Scr, ALT, SBP

*TyG index* triglyceride glucose index, *METS-IR* metabolic score for Insulin resistance, *TG* triglyceride, *HDL-C* high-density lipoprotein-cholesterol, *HOMA-IR* homeostatic model assessment of insulin resistance, *BMI* body mass index, *PIR* the ratio of family income to poverty, *CVD* cardiovascular disease, *Scr* serum creatinine, *SBP* systolic pressure, *BUN* blood urea nitrogen, *HbA1C* glycosylated hemoglobin, *FPG* fasting plasma glucose, *ALT* alanine transaminase, *HR* hazard ratio, *CI* confidence interval

*P < 0.05

**P < 0.01

***P < 0.001

*P* values in bold are < 0.05

### The detection of nonlinear relationships

**Fig. 4:**
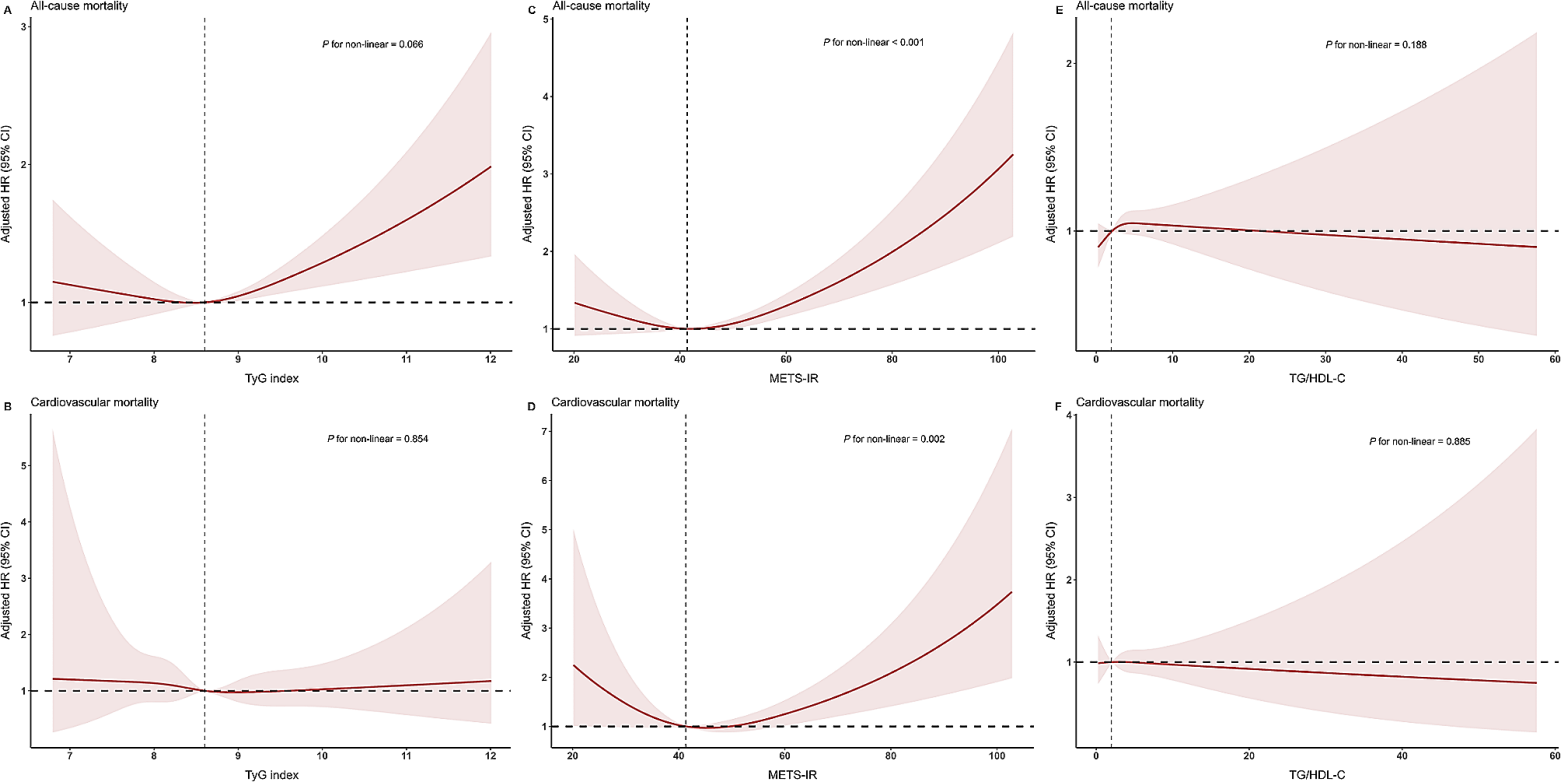
Association between TyG index and all-cause (**A**) and cardiovascular mortality (**B**) in the general population. Each hazard ratio was calculated with the TyG index level of 8.61 as reference. Association between METS-IR and all-cause (**C**) and cardiovascular mortality (**D**) in the general population. Each hazard ratio was calculated with the METS-IR level of 41.33 as reference. Association between TG/HDL-C and all-cause (**E**) and cardiovascular mortality (**F**) in the general population. Each hazard ratio was calculated with the TG/HDL-C level of 1.98 as reference. Association between HOMA-IR and all-cause (**G**) and cardiovascular mortality (**H**) in the general population. Each hazard ratio was calculated with the HOMA-IR level of 2.49 as reference. All-cause mortality was adjusted for age, gender, race, BMI, PIR, education, drinking, smoking, CVD, Scr, SBP, BUN, hypertension. Cardiovascular mortality was adjusted for age, gender, race, BMI, PIR, education, drinking, smoking, HbA1C, FPG, Scr, ALT, SBP. The solid line and red area represent the estimated values and their corresponding 95% CIs, respectively. *TyG index* triglyceride glucose index, *METS-IR* metabolic score for Insulin resistance, *TG* triglyceride, *HDL-C* high-density lipoprotein-cholesterol, *HOMA-IR* homeostatic model assessment of insulin resistance, *BMI* body mass index, *PIR* the ratio of family income to poverty, *CVD* cardiovascular disease, *Scr* serum creatinine, *SBP* systolic pressure, *BUN* blood urea nitrogen, *HbA1C* glycosylated hemoglobin, *FPG* fasting plasma glucose, *ALT* alanine transaminase, *HR* hazard ratio, *CI* confidence interval

Considering that multivariate Cox proportional risk analyses indicated a potential nonlinear relationship between the three indexes and all-cause and cardiovascular mortality, we employed restricted cubic splines analysis to further investigate this correlation. Figure 4 shows adjusted restricted cubic spline plots of nonlinear associations between METS-IR and all-cause (Fig. 4C, nonlinear *P* < 0.001) and cardiovascular mortality (Fig. 4D, nonlinear *P* = 0.002). Although there was no significant nonlinear association between TyG index, TG/HDL-C, HOMA-IR and mortality, we chose the point at which the adjusted HR was 0 as the "inflection point". We fitted the associations between the four indexes and mortality using a standard Cox proportional risk regression model and a two-segmented Cox proportional risk regression model (Table 5). Although we incorporated different variables in exploring the inflection points for all-cause and cardiovascular mortality, through two-segmented Cox proportional risk regression models, we found that these four IR indexes had the same inflection points for all-cause mortality as they did for cardiovascular mortality. The inflection points of the TyG index, METS-IR, TG/HDL-C and HOMA-IR were 8.61, 41.33, 1.98 and 2.49, respectively. When the TyG index was higher than 8.61, for each unit increase in TyG index, the adjusted HR for all-cause mortality increased by 25.7% (HR 1.257; 95% CI 1.108, 1.426, *P* for log-likelihood ratio = 0.005), while there was no significant correlation with cardiovascular mortality. When METS-IR was higher than 41.33, for each unit increase in METS-IR, the adjusted HR for all-cause mortality and cardiovascular mortality increased by 1.9 and 2.8%, respectively (HR 1.019; 95% CI 1.011, 1.026 and HR 1.028; 95% CI 1.014, 1.043, respectively, *P* for log-likelihood ratio < 0.05). In the two-segmented Cox proportional risk regression models of TG/HDL-C, there was no significant correlation with either all-cause mortality or cardiovascular mortality (*P* for log-likelihood ratio > 0.05). Adjusted HR for all-cause mortality increased by 1.5% per unit increase in HOMA-IR when HOMA-IR was higher than 2.49 (HR 1.015; 95% CI 1.010, 1.020, *P* for log-likelihood ratio < 0.001), while there was no correlation with cardiovascular mortality.

**Table 5:** Threshold effect analysis of TyG index, METS-IR, TG/HDL-C and HOMA-IR on all-cause and cardiovascular mortality, NHANES 2001–2018

TyG index	Adjusted HR (95% *Cl*), *P*-Value	METS-IR	Adjusted HR (95% *Cl*), *P*-Value	TG/HDL-C	Adjusted HR (95% *Cl*), *P*-Value	HOMA-IR	Adjusted HR (95% *Cl*), *P*-Value
All-cause mortality							
Fitting by the standard linear model	1.130 (1.039–1.230) **0.004**	Fitting by the standard linear model	1.015 (1.008–1.022) < **0.001**	Fitting by the standard linear model	1.003 (0.991–1.014) 0.630	Fitting by the standard linear model	1.016 (1.011–1.021) < **0.001**
Fitting by the two-piecewise linear model		Fitting by the two-piecewise linear model		Fitting by the two-piecewise linear model		Fitting by the two-piecewise linear model	
Inflection point	8.61	Inflection point	41.33	Inflection point	1.98	Inflection point	2.49
TyG index < 8.61	0.925 (0.678–1.262) 0.622	METS-IR < 41.33	0.972 (0.950–0.997) **0.030**	TG/HDL-C < 1.98	0.879 (0.686–1.127) 0.310	HOMA-IR < 2.49	0.982 (0.833–1.157) 0.826
TyG index ≥ 8.61	1.257 (1.108–1.426) < **0.001**	METS-IR ≥ 41.33	1.019 (1.011–1.026) < **0.001**	TG/HDL-C ≥ 1.98	1.000 (0.989–1.012) 0.979	HOMA-IR ≥ 2.49	1.015 (1.010–1.020) < **0.001**
*P* for Log-likelihood ratio	**0.005**	*P* for Log-likelihood ratio	** < 0.001**	*P* for Log-likelihood ratio	0.630	*P* for Log-likelihood ratio	** < 0.001**
Cardiovascular mortality							
Fitting by the standard linear model	0.959 (0.765–1.201) 0.713	Fitting by the standard linear model	1.018 (1.004–1.032) **0.012**	Fitting by the standard linear model	0.996 (0.972–1.021) 0.748	Fitting by the standard linear model	1.008 (0.999–1.018) < **0.001**
Fitting by the two-piecewise linear model		Fitting by the two-piecewise linear model		Fitting by the two-piecewise linear model		Fitting by the two-piecewise linear model	
Inflection point	8.61	Inflection point	41.33	Inflection point	1.98	Inflection point	2.49
TyG index < 8.61	1.025 (0.521–2.016) 0.943	METS-IR < 41.33	0.964 (0.915–1.015) 0.162	TG/HDL-C < 1.98	0.953 (0.595–1.525) 0.839	HOMA-IR < 2.49	0.964 (0.691–1.345) 0.830
TyG index ≥ 8.61	1.042 (0.758–1.432) 0.801	METS-IR ≥ 41.33	1.028 (1.014–1.043) < **0.001**	TG/HDL-C ≥ 1.98	0.995 (0.973–1.018) 0.687	HOMA-IR ≥ 2.49	1.010 (1.002–1.018) < **0.001**
*P* for log-likelihood ratio	0.706	*P* for log-likelihood ratio	**0.022**	*P* for log-likelihood ratio	0.735	*P* for log-likelihood ratio	0.124

All-cause mortality was adjusted for age, gender, race, BMI, PIR, education, drinking, smoking, CVD, Scr, SBP, BUN, hypertension

Cardiovascular mortality was adjusted for age, gender, race, BMI, PIR, education, drinking, smoking, HbA1C, FPG, Scr, ALT, SBP

*TyG index* triglyceride glucose index, *METS-IR* metabolic score for Insulin resistance, *TG* triglyceride, *HDL-C* high-density lipoprotein-cholesterol, *HOMA-IR* homeostatic model assessment of insulin resistance, *BMI* body mass index, *PIR* the ratio of family income to poverty, *CVD* cardiovascular disease, *Scr* serum creatinine, *SBP* systolic pressure, *BUN* blood urea nitrogen, *HbA1C* glycosylated hemoglobin, *FPG* fasting plasma glucose, *ALT* alanine transaminase, *HR* hazard ratio, *CI* confidence interval

*P* values in bold are < 0.05

### Stratified analyses

**Fig. 5:**
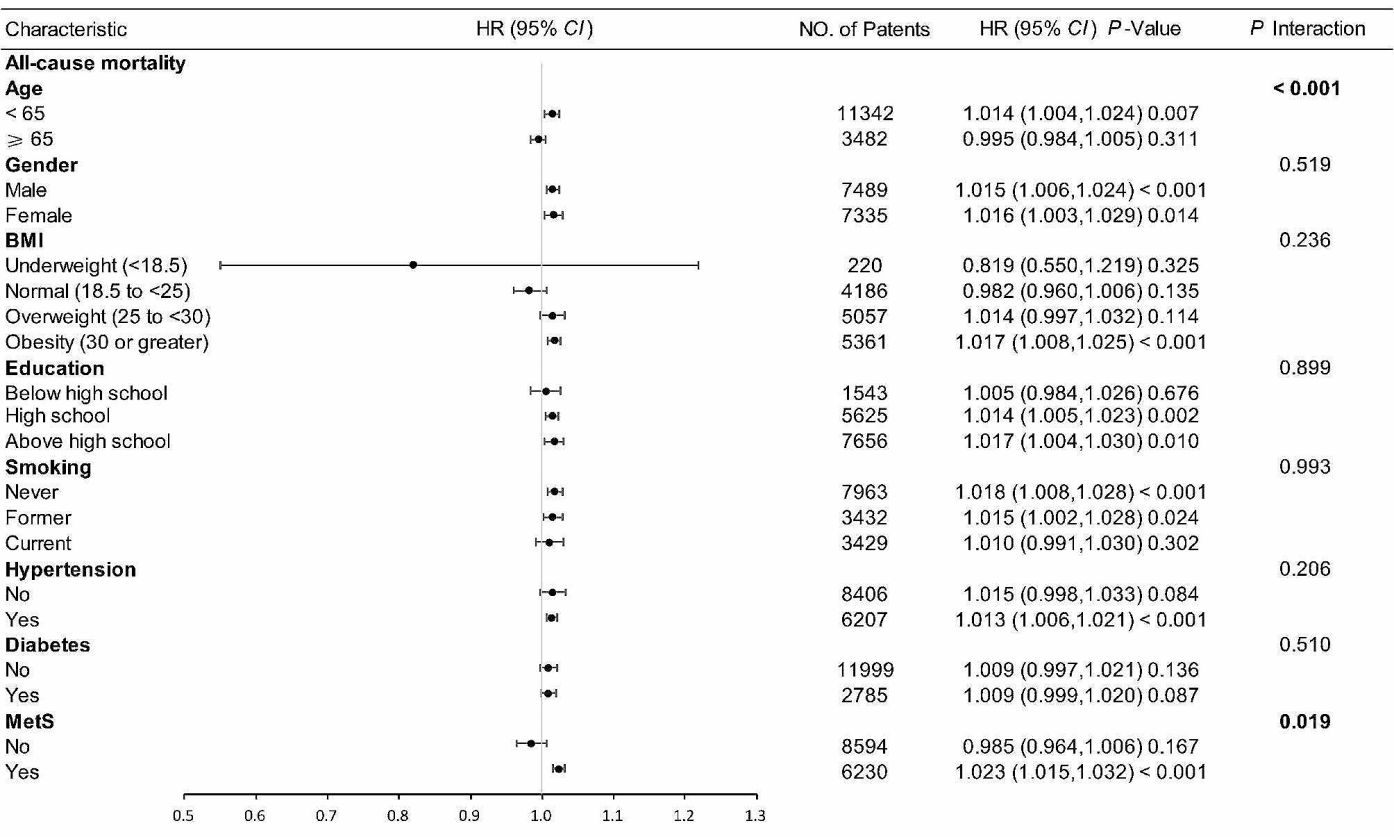
Subgroup analysis of the association between METS-IR and all-cause mortality. Adjusted for age, gender, race, BMI, PIR, education, drinking, smoking, CVD, Scr, SBP, BUN, hypertension, except the subgroup factors themselves. *METS-IR* metabolic score for insulin resistance, *BMI* body mass index, *PIR* the ratio of family income to poverty, *CVD* cardiovascular disease, *Scr* serum creatinine, *SBP* systolic pressure, *BUN* blood urea nitrogen, *MetS* metabolic syndrome, *HR* hazard ratio, *CI* confidence interval

**Fig. 6:**
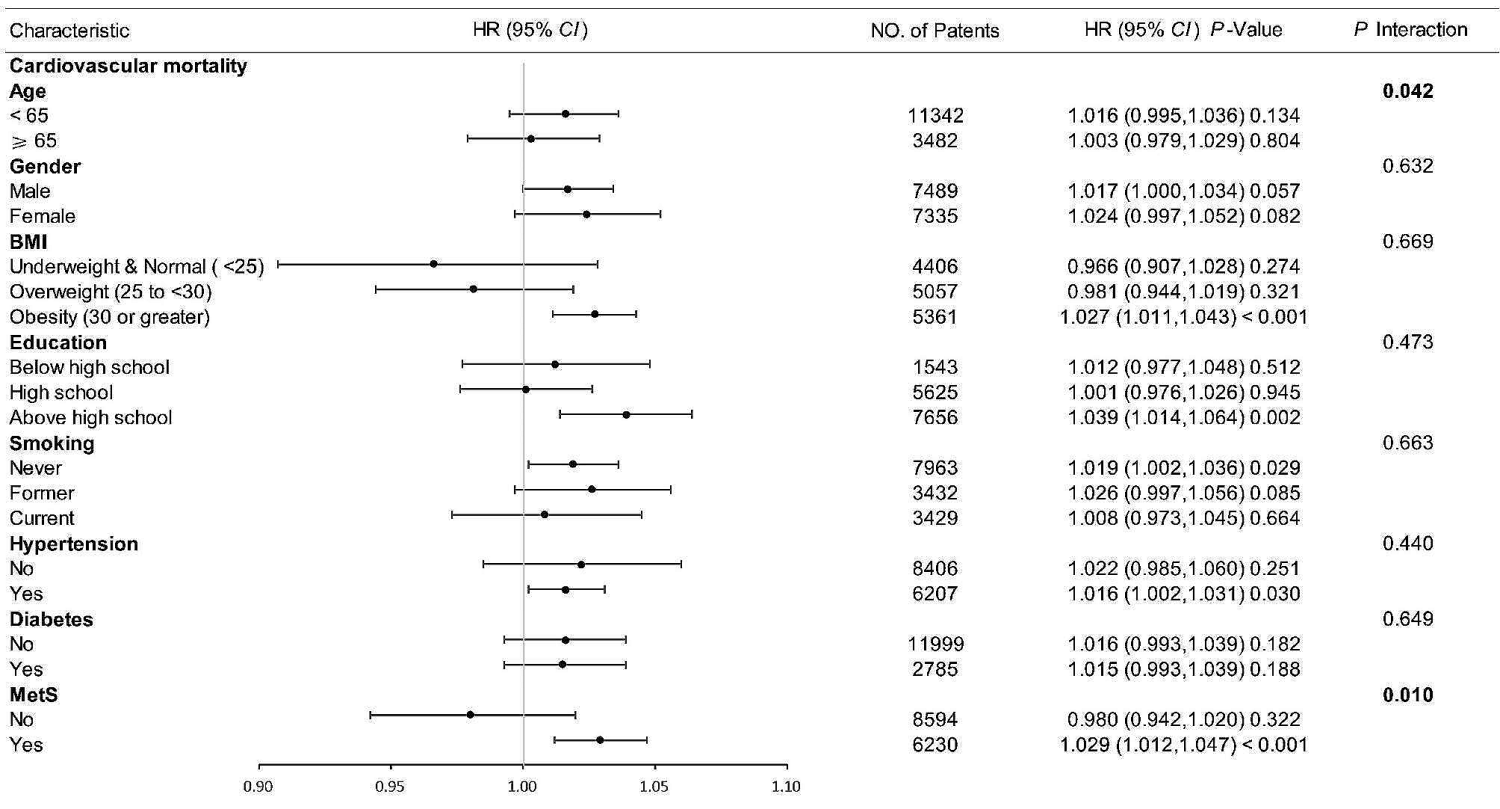
Subgroup analysis of the association between METS-IR and cardiovascular mortality. Adjusted for age, gender, race, BMI, PIR, education, drinking, smoking, HbA1C, FPG, Scr, ALT, SBP, except the subgroup factors themselves. *METS-IR* metabolic score for insulin resistance, *BMI* body mass index, *PIR* the ratio of family income to poverty, *HbA1C* glycosylated hemoglobin, *FPG* fasting plasma glucose, *Scr* serum creatinine, *ALT* alanine transaminase, *SBP* systolic pressure, *MetS* metabolic syndrome, *HR* hazard ratio, *CI* confidence interval

As shown in Figs. 5 and 6, to further assess the effect of METS-IR on outcome indicators, stratification was performed according to age, sex, BMI, education, smoking, hypertension, diabetes, and metabolic syndrome. There were no significant interactions in any of the subgroups except for the age subgroup (age subgroup: all-cause mortality: interaction *P* < 0.001, cardiovascular mortality: interaction *P* = 0.044) (other subgroups: all-cause mortality: interaction *P* = 0.241–0.937, cardiovascular mortality, interaction *P* = 0.363–0.679). METS-IR was strongly associated with all-cause mortality in patients < 65 years of age (HR (95% CI) 1.014 (1.004, 1.024), *P* = 0.008), but not in patients aged ≥ 65 years (HR (95% CI) 0.994 (0.983, 1.004), *P* = 0.247).

## Discussion

To our knowledge, this is the first study comparing the effect of TyG index with other surrogate indexes of IR (METS-IR, TG/HDL-C, HOMA-IR) on mortality in a large cohort. Our study found that TyG index, METS-IR, TG/HDL-C and HOMA-IR were positively correlated with age, BMI, blood pressure, HbA1C, FINS, FPG, TG, UA, BUN, ALT, GGT, serum potassium, WBC and PLT, and negatively correlated with HDL-C. This correlation may be attributed to the presence of traditional CVD risk factors, which have been reported previously [27]. In addition, in contrast to the previous approach of screening variables based on clinical experience alone, we combined the results of Boruta’s algorithm with traditional risk factors to include 13 clinical indicators in the multifactorial Cox regression analyses of all-cause mortality and cardiovascular mortality, respectively. After multivariate Cox regression and restricted cubic splines analysis, we unexpectedly found that the METS-IR performed better than the other three indexes, with significant correlations with both all-cause mortality and cardiovascular mortality, and both showed nonlinear associations that were similar to a “U-shaped” association. Furthermore, we conducted a threshold effect analysis and determined that the inflection point for METS-IR in both all-cause mortality and cardiovascular mortality was 41.33. After adjusting for confounders, each unit increase in the baseline METS-IR was associated with an increase in all-cause and cardiovascular mortality by 1.9 and 2.8%, respectively, for those with the baseline METS-IR above the inflection point. In contrast, TyG index and HOMA-IR were significantly associated with all-cause mortality only, whereas TG/HDL-C was not significantly associated with either mortality. Our study suggests that the METS-IR can be used as a predictor of all-cause and cardiovascular mortality in the general population when combined with important features derived from Boruta algorithm screening.

A large number of studies have been conducted on the relationship between TyG index and mortality in different populations. Chen et al. observed that there was a significant association between TyG index and all-cause and cardiovascular mortality in the general population, and that this association was most pronounced in the 45–64 year age group [13]. Liu et al. found that elevated TyG index can independently predict the mortality of ischemic stroke patients under 65 years old at 3 and 12 months [28]. And in patients with CHD, TyG index was shown to be positively associated with future cardiovascular events, suggesting that TyG index may be a useful predictor of clinical outcomes [29]. In a recent study, Zhang et al. demonstrated a U-shaped association between baseline TyG index and all-cause and cardiovascular disease mortality in patients with diabetes or prediabetic cardiovascular disease [4]. In addition, as early as 2010 Guerrero et al. found that the TyG index showed a high correlation with hyperinsulinemic-euglycemic clamp technique in IR assessment, with significant sensitivity and specificity [30]. Thus, although the exact biological mechanisms underlying the correlation between TyG index and mortality are unknown, the key pathways involved may be related to IR. In conclusion, the role of TyG index in the assessment of IR has been widely recognized and is regarded as a valuable indicator.

On the other hand, alternative estimates of insulin action have been widely used to study the relationship between IR and various clinical syndromes, and this has led to the development of a variety of IR replacement indexes, among which the METS-IR and TG/HDL-C are included. When calculating METS-IR, in addition to requiring fasting glucose and triglycerides, BMI and HDL-C are also needed. It has been shown that METS-IR is significantly associated with fat content in the liver and pancreas, and ectopic fat accumulation in muscle and liver tissues has been suggested as a mechanism for the development of IR [31, 32]. METS-IR, as a novel scoring system for screening insulin sensitivity, can effectively identify individuals at high risk of insulin resistance-associated pathological changes, thus saving costs associated with fasting insulin measurements [33]. It has been shown that the diagnostic performance of METS-IR was significantly higher than the TyG index and TG/HDL ratio, but did not differ from the TyG-BMI index [34]. Ramírez et al. assessed the relationship between obesity and IR by evaluating the abdominal volume index (AVI) and body fat index (BAI) using three risk scales (TyG index, METS-IR and TG/HDL-C), concluding that only in the case of the METS-IR, the AVI and BAI proved to be of value in the prediction of IR [35]. Furthermore, there have also been a number of studies on TG/HDL-C. One study of 449 healthy individuals found that TG/HDL provided an estimate of insulin sensitivity that was as accurate as using FPG and insulin concentration measures, and that the greater the TG/HDL ratio, the more insulin tolerant the patient was [36]. Karelis et al. further supported the significant correlation between TG/HDL-C and indicators of insulin action in a specific population by studying 131 overweight and obese menopausal women [37]. HOMA-IR, on the other hand, has been validated in the general population as early as 1999 and can be used as a common indicator for assessing IR [38]. Meta-analysis of prostate cancer patients by Somayeh et al. showed that HOMA-IR levels were positively correlated with fasting insulin levels, especially in patients older than 65 years [39]. However, due to the heterogeneity of study populations, sample sizes and follow-up times, the predictive effect of the four IR replacement indexes (TyG index, METS-IR, TG/HDL and HOMA-IR) on mortality in a large sample of the general population remains uncertain.

This study explores the predictive value of four IR replacement indexes (TyG index, METS-IR, TG/HDL and HOMA-IR), in relation to mortality in the general population through multivariate Cox proportional risk analysis, incorporating the results of Boruta’s algorithm. The Boruta algorithm is a Random Forest-based feature selection method that identifies the most important features by comparing the Z-value of each feature with the Z-value of the “shaded features”. The Z-value of each attribute is obtained from the Random Forest model at each iteration by copying all the real features and destroying them sequentially, and the Z-value of the shadow is created by destroying the real features randomly. A feature is considered “important” if the Z-value of the real feature is greater than the maximum Z-value of the shaded feature in multiple independent trials [40, 41]. In contrast to previous multivariate regression analyses, which were based on clinical significance and experience, we evaluated the impact of the IR replacement indexes on all-cause and cardiovascular mortality more accurately by combining the most relevant characteristics of the dependent variable with the characteristics screened by Boruta’s algorithm. In our study, we found that among the four indexes, only METS-IR was significantly correlated with all-cause mortality and cardiovascular mortality, and all of them showed non-linear associations similar to a “U-shape”. Further stratified analysis of METS-IR showed significant interactions with age. Specifically, in the general population, significant associations between METS-IR levels and all-cause and cardiovascular mortality were observed mainly in the nonelderly population aged < 65 years. Exposure to insulin resistance resulting from the same disease duration may cause more severe complications in younger patients compared with older adults [13]. This is in part consistent with the study by Liu and Sharif et al. [28, 42].

## Strengths and limitations

This study has several advantages. First, we pioneered the application of the Boruta algorithm to select the inclusion factors for multifactorial Cox regression on the basis of a large sample of data, which improved the confidence and accuracy of the study. In addition, our study confirmed the association between METS-IR and all-cause and cardiovascular mortality in the general population, which mainly existed in those aged < 65 years. This not only provides a theoretical basis for the application of METS-IR in non-elderly populations, but also provides new clues for research in the field of IR replacement indexes.

It is also important to point out some limitations of this study. First, this was an observational study and could not prove a cause-and-effect relationship between METS-IR and mortality. Second, relying only on self-reported data from the NHANES questionnaire to obtain smoking, alcohol consumption and comorbidities may have memory bias and subjective bias. Third, the IR replacement index should change dynamically. However, due to cost and other limitations, we were only able to obtain baseline data. Finally, the findings are based only on survey data from the general population in the United States, so careful consideration is needed when generalizing the results to other races and populations. Future studies should be conducted with these limitations in mind to further deepen understanding in this area.

## Conclusion

This study retrospectively analyzed 14,653 participants from NHANES to compare the mortality-predicting effects of four IR replacement indexes (TyG index, METS-IR, TG/HDL and HOMA-IR) in the population. The results of the study showed that the METS-IR was significantly associated with all-cause and cardiovascular mortality in the general population, especially in those under 65 years of age.

## Data Availability

Data supporting the results of this study are available from the first author.

## Abbreviations

ATP 3: Adult treatment third edition
AVI: Abdominal volume index
ALT: Alanine transaminase
AST: Aspartate transaminase
BAI: Body fat index
BMI: Body mass index
BUN: Blood urea nitrogen
CDC: Centers for disease control and prevention
CHD: Coronary heart disease
CHF: Congestive heart failure
CI: Confidence interval
CVD: Cardiovascular disease
DBP: Diastolic blood pressure
FINS: Fasting serum insulin
FPG: Fasting plasma glucose
GGT: Gamma glutamyl transferase
HbA1C: Glycosylated hemoglobin
HDL-C: High-density lipoprotein cholesterol
HOMA-IR: Homeostasis model assessment of insulin resistance
HR: Hazard ratio
IR: Insulin resistance
LDH: Lactate dehydrogenase
LDL-C: Low-density lipoprotein cholesterol
MEC: Mobile examination center
METS-IR: Metabolic score for insulin resistance
MetS: Metabolic syndrome
MI: Myocardial infarction
NCEP: National cholesterol education program
NCHS: National center for health statistics
NDI: National death index
NHANES: National health and nutrition examination survey
PIR: Poverty income ratio
PLT: Blood platelet
SBP: Systolic blood pressure
Scr: Serum creatinine
SD: Standard deviation
TC: Total cholesterol
TBil: Total bilirubin
TG: Triglycerides
TyG index: Triglyceride glucose index
WC: Waist circumference
WBC: White blood cells
